# Discovery of a Lineage of Soil-Dwelling *Medetera* Species with Multi-Coloured Eyes in Southern Europe (Diptera: Dolichopodidae)

**DOI:** 10.3390/insects13111012

**Published:** 2022-11-02

**Authors:** Marc Pollet, Rui Andrade, Ana Gonçalves, Piluca Álvarez Fidalgo, José Luis Camaño Portela, Frédéric Belin, Jonas Mortelmans, Andreas Stark

**Affiliations:** 1 Research Institute for Nature and Forest (INBO), B-1000 Brussels, Belgium; 2 Operational Directory Taxonomy and Phylogeny, Entomology, Royal Belgian Institute of Natural Sciences (RBINS), B-1000 Brussels, Belgium; 3 Independent Researcher, 4050-145 Porto, Portugal; 4 Graduate Program in Entomology, Instituto Nacional de Pesquisas da Amazônia—INPA, Manaus CEP 69067-375, Amazonas, Brazil; 5 Independent Researcher, 28002 Madrid, Spain; 6 Escuela de Ingeniería Industrial, University of Vigo, 36310 Vigo, Spain; 7 Independent Researcher, 06220 Golfe-Juan, France; 8 Independent Researcher, B-9000 Gent, Belgium; 9 Zentralmagazin Naturwissenschaftlicher Sammlungen, Martin-Luther-Universität Halle-Wittenberg, D-06108 Halle, Germany; 10 Senckenberg German Entomological Institute (SDEI) Müncheberg, D-15374 Müncheberg, Germany

**Keywords:** Mediterranean region, Iberian Peninsula, Sardinia, Corsica, eye pattern, phylogeny, ecology

## Abstract

**Simple Summary:**

Long-legged flies or Dolichopodidae are one of the most speciose fly families of the order Diptera. At present, dolichopodid faunas in the tropics still encompass a large undescribed fraction, but in Europe new species can also be found. Recently, two new species of *Medetera* with a conspicuous eye colour pattern were discovered in Portuguese samples. Increased subsequent collecting efforts in southwestern Europe added another five species, most of which also featured a distinct eye colour pattern. All seven species are described in this paper with information on their distribution and ecology. An identification key to males is also provided. All these species share genital and some non-genital characters which suggests that they belong to the same lineage within this genus. Barcoding data seem to confirm this, as does the ecological information. Indeed, contrary to many *Medetera* species which are confined to tree trunks where their larvae feed on all stages of bark beetles, several of the new species are mainly found on hard, often rocky, substrates also in open habitats.

**Abstract:**

Seven species in the genus *Medetera* (Diptera: Dolichopodidae) are described here: *Medetera aglaops* sp. nov., *Medetera corsicana* sp. nov., *Medetera gibbosipyga* sp. nov., *Medetera hispanica* sp. nov., *Medetera lusitana* sp. nov., *Medetera parva* sp. nov., and *Medetera rectipyga* sp. nov. They all originate from the wider Mediterranean region in southwestern Europe (Iberian Peninsula, southern France, Corsica, Sardinia). The most striking feature in most of the species is the bi- or multicolour pattern of the eyes, most conspicuous in *Medetera aglaops* sp. nov. and *Medetera lusitana* sp. nov. This character is shared by the male and female sex and is thus not regarded as Male Secondary Sexual Character. All species belong to the *Medetera apicalis* species group *sensu* Bickel and are closely related based on shared characters in the hypandrium and cercus. Interestingly, three species with and four species without the basal pair of anterodorsal and posterodorsal bristles on the mid tibia are represented. This suggests that this diagnostic character has less phylogenetic relevance than previously anticipated. Unlike most Palaearctic *Medetera*, at least five of the new species are mostly found on rocky substrates in dry biotopes with a sclerophyllous vegetation. This further supports their relationship with species of the *Medetera muralis* subclade *sensu* Pollet, Germann and Bernasconi.

## 1. Introduction

On a global scale, the ubiquitous *Medetera* Fischer von Waldheim, 1819 represents the third most speciose genus in Dolichopodidae (Diptera) with an apparent predominance in the Palaearctic [1]. In the latter realm this genus includes 190 species and three subspecies [2,3]. Overall, *Medetera* species range in size from very small (<2 mm) to rather large (>4.5 mm). All seven most recently described species from the western Palaearctic were detected in Switzerland [4,5]. However, this does not necessarily imply that only high altitude or latitude sites in the Palaearctic region, and Europe in particular, house yet undiscovered species. As such and based on current data and knowledge, overall this genus makes up a larger proportion of the dolichopodid fauna in northern and northwestern European countries as compared to southern ones [6]. However, these authors also reported 21 unidentified *Medetera* species from Portugal, accounting for 10% of the dolichopodid species registered for this country. Moreover, with 41 species, the entire genus represented even two times this proportion (i.e., 20.5%) in Portugal, which is three times higher than, e.g., Spain or Italy.

With respect to their biology, many *Medetera* can be found on tree trunks where their larvae live in galleries of scolytine beetles (Coleoptera: Curculionidae, Scolytinae) and feed on all stages of these bark beetles e.g., [7,8,9,10,11]. This holds especially true for the species groups of *M. apicalis*, *M. crassivenis*, and *M. signaticornis–pinicola sensu* Bickel, with the former two preferring deciduous and the latter one coniferous trees [12,13]. In the Holarctic realm, one lineage in particular deviates from this pattern by occurring, often in large numbers, on other hard, mostly vertical, substrates and also on bare or sparsely vegetated sandy soils, i.e., the *M. diadema–veles* species group *sensu* Bickel. This clade also includes the psammophilous species with one pair of scutellar bristles that were previously treated as a separate subgenus *Oligochaetus* Mik, 1878. Another lineage, the *M. muralis* species group *sensu* Pollet, Germann and Bernasconi (a subclade of the *M. apicalis* species group, see [13]) on the other hand, is also regularly encountered on hard substrates (rocks, walls).

**Figure 1:**
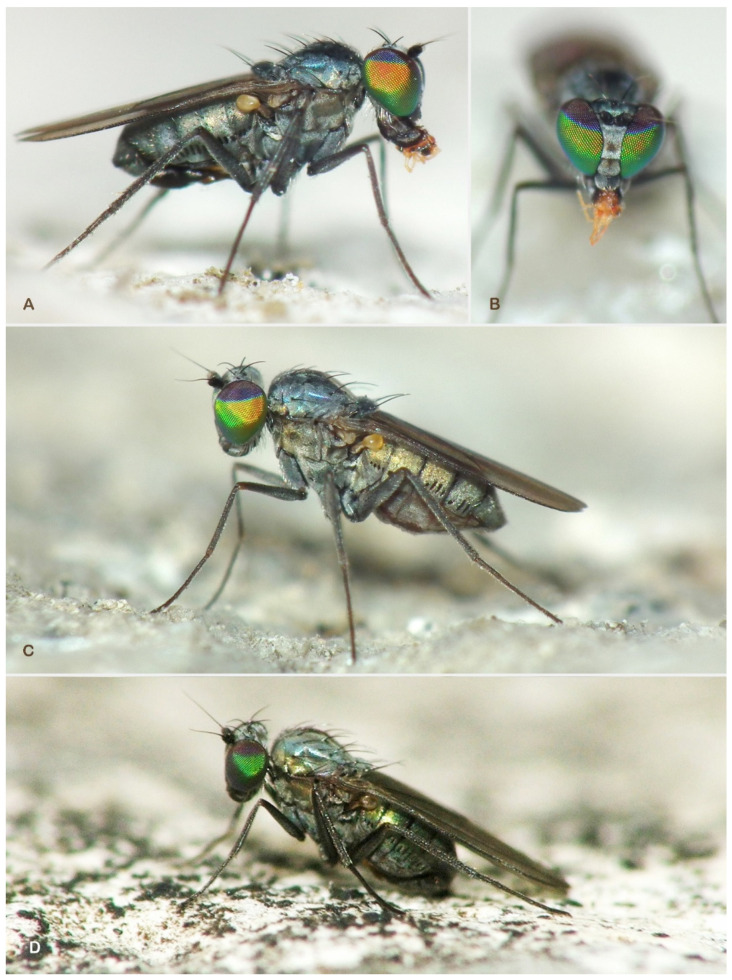
*Medetera* species with bi-coloured eyes from Portugal. (**A**) *Medetera aglaops* sp. nov., male (lateral view); (**B**) *Medetera aglaops* sp. nov., male (frontal view); (**C**) *Medetera aglaops* sp. nov., female (lateral view); (**D**) *Medetera lusitana* sp. nov. (male) (photos: Rui Andrade).

During the examination of Portuguese dolichopodid samples [6] the first author (MP) noticed two *Medetera* species with a remarkable eye pattern: the lower 2/3 of the eye featured a usual red base colour with a brilliant green reflection while the upper 1/3 was conspicuously and contrastingly dark violet (Figure 1). Increased attention during the collecting and continued processing of additional samples from the Iberian Peninsula and the Mediterranean region added another five undescribed *Medetera* species that appeared to be related. Moreover, some of these species also showed a distinct eye colour pattern.

In the present paper, seven southern European *Medetera* species are described, with information on their distribution and ecology, and a key to the males is provided. The significance of some morphological characters and the phylogenetic position of the species is discussed as well as issues with the verification of *Medetera* species identity and reasons for their surprisingly late discovery.

## 2. Materials and Methods

In addition to the 761 Portuguese samples [6], another 21.093 dolichopodid samples from 20 European countries were inspected for any multi-colour eyed *Medetera* species by the first author. None of the over 20,000 samples from 18 European countries that had been examined prior to 2018 contained any of these species, but recently acquired samples from southern Europe (Portugal, Spain, southern France, Corsica, Sardinia) did surprisingly often. Most of the samples were acquired thanks to special collecting efforts by Diptera workers and other people (see co-authors and Acknowledgements). No additional material from museum collections was examined. Actually, we have no knowledge of any dolichopodid collections in Portugal or Spain that might contain some of these *Medetera* species. Moreover, the probability of finding these species in the collections of the Muséum national d’Histoire naturelle (MNHN, Paris, France) was considered low. It must be taken into account, however, that in (dry) mounted specimens, the eye colour reflection fades away over time, while the colour itself might slightly change in specimens stored in alcohol solution.

Specimens from Corsica were collected in the frame of the most recent edition of the La Planète Revisitée (Our Planet Reviewed) series of surveys [14], organised in several undersampled biodiversity hotspots around the world since 2007 by, e.g., the MNHN (see [15]). The terrestrial part of this survey (along with the marine counterpart) focused on the diversity of—often poorly studied—invertebrates, including Arachnida, Mollusca as well as all major insect orders (i.e., Coleoptera, Diptera, Hymenoptera, Lepidoptera, Orthoptera, Hemiptera), fungi, and lichens [16]. The main objective was a comprehensive inventory of species in a series of localities in order to improve knowledge of the taxonomy and chorology, and to better understand community structures and their interactions within ecosystems. Wherever feasible, it aimed to contribute to a better management of the territory through assessments and monitoring. Species records will ultimately be disclosed and distributed in the frame of the Inventaire National du Patrimoine Naturel [17] and, in the case of this specific survey, via the portal of the MNHN collections [18]. Specimens from Sardinia were gathered during a CONECOFOR survey on the island conducted by G.L. Nardi and colleagues of the Centro Nazionale Carabinieri Biodiversità “Bosco Fontana” (CNCB) [19,20,21,22].

Species descriptions were based on a representative number of specimens of each species, all stored in alcohol solution. Features where an effect was expected due to the wet condition of the specimen (e.g., colour and pollinosity of face, frons, eyes) were described on the basis of temporarily dried specimens. A total of 173 character states were scored in each species, with 35, 61, and 77 related to the head, thorax/abdomen/wing and legs, respectively. This allowed us to determine the most reliable and consistent decisive diagnostic features that were subsequently applied in the identification key (see further). Photos of the general habitus and head of each species were produced by the last author (AS). The hypopygium and separate genital appendages of each species were drawn using a camera lucida. In each species, at least the hypopygium (in lateral view) and the hypandrium (in ventral view) are illustrated here. In describing the hypopygium, ‘dorsal’ and ‘ventral’ refers to the morphological position prior to genitalic rotation and flexion. Thus, in the drawings showing a lateral view of the hypopygium, the top is morphologically ventral, while the bottom is dorsal. Biometrics were based on 5 specimens of each gender in each species, if available or unless otherwise mentioned, and included: (i) face width, (ii) body length, (iii) wing length (= distance between basis of basicosta and wing apex), (iv) relative wing width, (v) proximal versus apical section of vein M_1_, (vi) proximal versus apical section of vein CuA_1_, (vii) CuA_x_ ratio (= crossvein dm-cu versus apical section of vein M_1_) and (viii) relative lengths ratio of femur, tibia, and tarsomeres of each leg. The latter relative lengths were recalculated so that the shortest leg part represented a value of “1″. All values given in this paper are ranges or average values, unless otherwise mentioned. Palp and proboscis size were compared to the eye size, measured as the vertical diameter (from about ocellar tubercle to the lower eye margin). The surstylus length was defined as the distance between the insertion place of the apicoventral epandrial setae and the surstylus apex.

COI barcode sequences of ca. 500 b.p. lengths were obtained from specimens of four of the new species. In addition, barcodes of 20 specimens belonging to 8 other *Medetera* and 2 *Thrypticus* species were mined from the BOLD Systems repository [23] (see Discussion). All sequences were aligned using MAFFT [24]. Evolutionary distances were computed by use of the p-distance method [25], with pairwise deletion.

**Figure 2:**
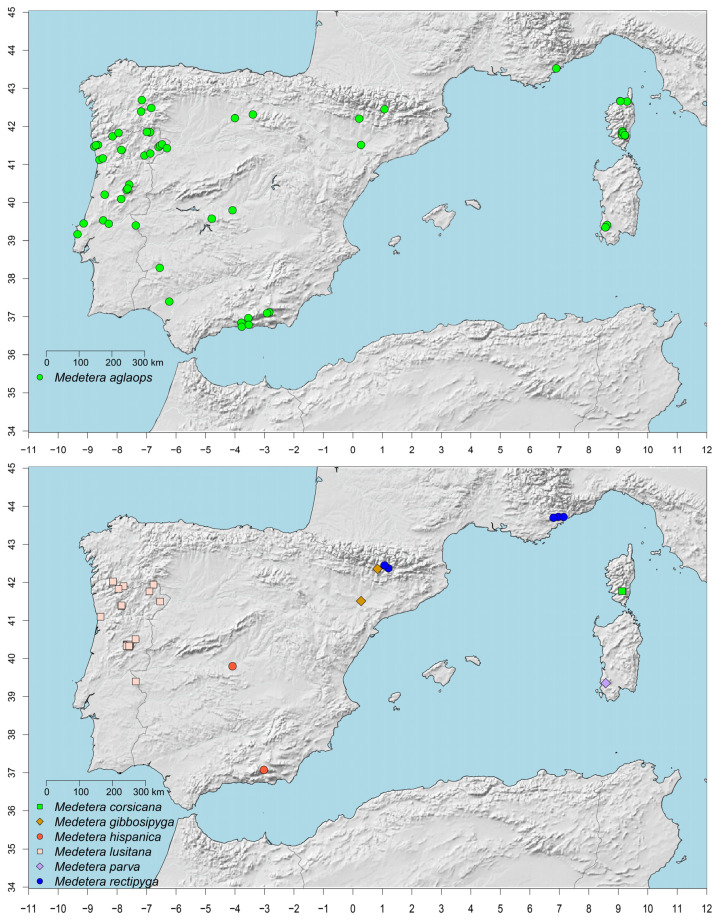
Distribution of new *Medetera* species in southern Europe.

Capture locations of the species are indicated in Figure 2. Each location was positioned on the map as accurately as possible, based on the information available. In two cases (Alfaz del Pi and Elguera, both in Spain) that lacked latitude and longitude data, the symbol in Figure 2 is shown in the centre of the corresponding locality/location which might not necessarily correspond exactly with the actual collecting site.

The general morphological terminology followed [26], while [27] was used for male genitalia. The following abbreviations were used: ac: acrostichal bristles; ad: anterodorsal; ant pprn: anterior postpronotal (= humeral *sensu* Parent [28]); ap: apical; apv: apicoventral; av: anteroventral; bas pprn: basal postpronotal (= posthumeral *sensu* Parent); bv: basoventral; dc: dorsocentral bristle pairs; ds: dorsal; MSSC(s): male secondary sexual character(s); npl: notopleural; pal: postalar; pd: posterodorsal; psut ial: presutural intra-alar (= presutural *sensu* Parent); pv: posteroventral; S: abdominal sternite; spal: supra-alar; sut ial: sutural intra-alar (= sutural *sensu* Parent); ta: tarsomere, 1–5 in the descriptions of tarsi refers to basal (1) to apical (5) tarsomeres; T: abdominal tergite; vt: ventral; I, II, III refers to fore, mid and hind leg; 1–6 in the descriptions of abdominal segments (tergites/sternites) refers to basal (1) to caudal (6) segments.

Other abbreviations used: BPT: blue pan traps, HC: collected by hand, HT: holotype, MT: Malaise trap, PT: pan traps, RsPT: pink pan traps, SW: collected by sweepnet, WPT: white pan traps, YPT: yellow pan traps. MAPC: private collection of Marc A. A. Pollet, Welle, Belgium; MNCN: Museo Nacional de Ciencias Naturales, Madrid, Spain; MNHN: Muséum national d’Histoire naturelle, Paris, France; MZUR: Museo di Zoologia Sapienza Università di Roma, Rome, Italy; RBINS: Royal Belgian Institute of Natural Sciences, Brussels, Belgium.

Species records are given in the following format (if available): “COUNTRY: m#, f#, province (or equivalent administrative division), locality, location (brief habitat description) (site code), latitude, longitude, altitude, sampling date (start)—sampling date (end), sampling method, collector (survey, if relevant)—sample cd: sample code [collection]”. Locality is defined as the most accurate administrative geographical entity (e.g., village, city), and location as most relevant non-administrative entity (e.g., area, river, nature reserve). Identical information in successive records is only given in the first record and not repeated in the following ones. Data including an often more detailed sampling site description are also available as a dataset in GBIF (https://doi.org/10.15468/s8c7n9). All specimens examined were stored in 75% alcohol.

## 3. Results

### 3.1. Species Descriptions

Zoobank registration: urn:lsid:zoobank.org:pub:7B7E476D-184B-4519-A987-8AFF163CDD3D

**Dolichopodidae** Latreille, 1809

**Medeterinae** Lioy, 1863–1864

***Medetera*** Fischer von Waldheim, 1819

**Figure 3:**
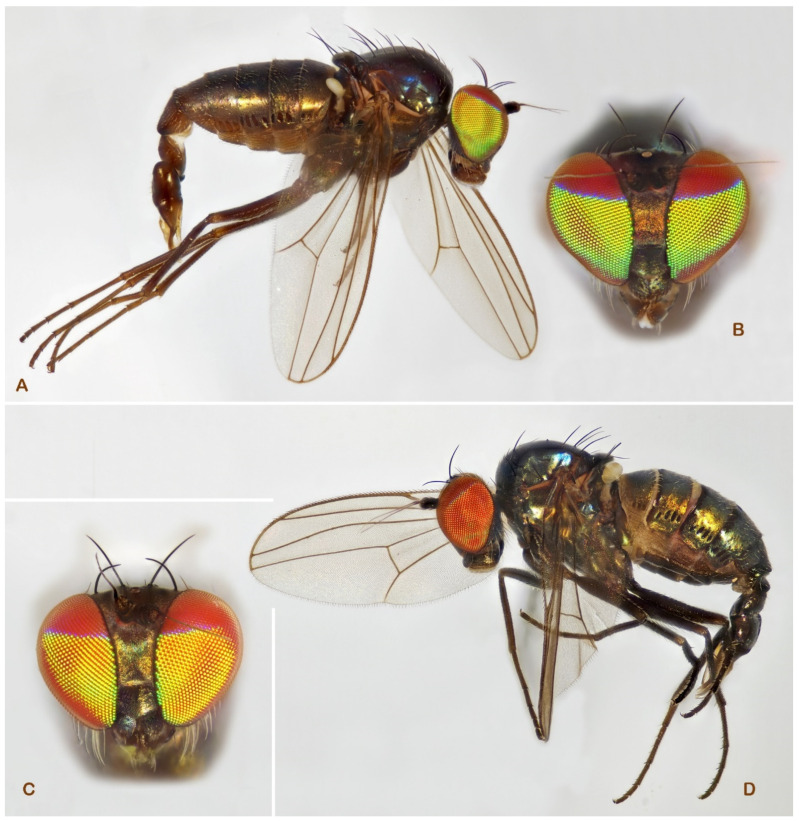
*Medetera aglaops* sp. nov. (**A**–**C**) Portuguese specimens. (**A**) male, habitus; (**B**) male, face; (**C**) female, face; (**D**) Corsican specimen, male, habitus (photos: Andreas Stark).

**Figure 4:**
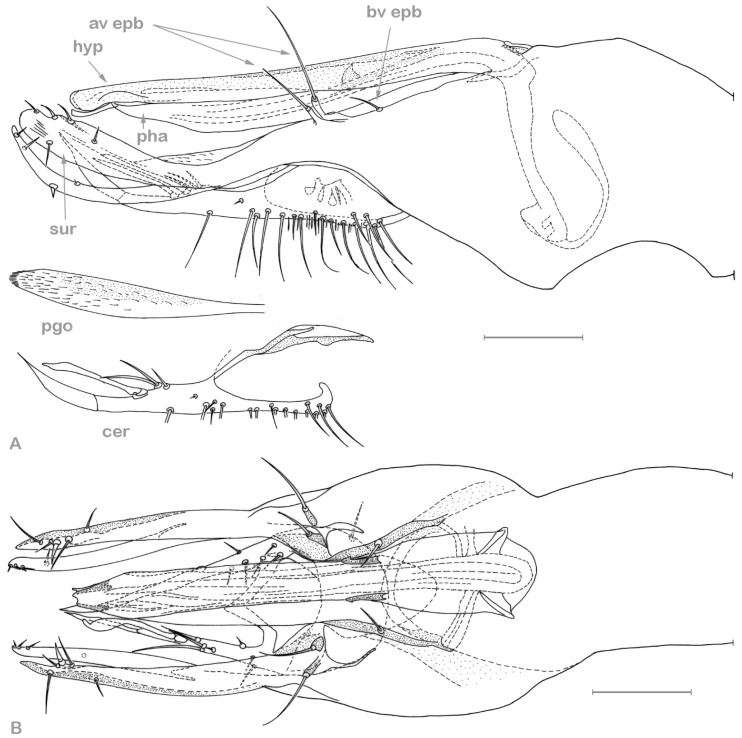
*Medetera aglaops* sp. nov., Portuguese specimen (**A**) hypopygium, lateral view; (**B**) hypopygium, ventral view. Av epb: apicoventral epandrial bristles, bv epb: basoventral epandrial bristles, cer: cercus, hyp: hypandrium, pha: phallus, pgo: postgonite, sur: surstylus. Scale = 0.1 mm.

**Figure 5:**
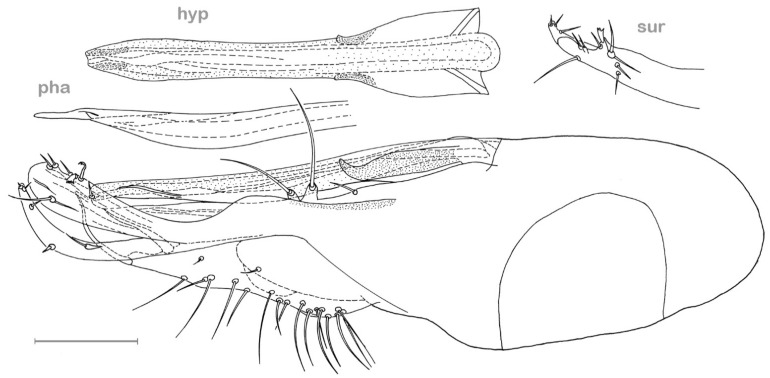
*Medetera aglaops* sp. nov., Corsican specimen, hypopygium, lateral view. Hyp: hypandrium, pha: phallus, sur: apical part of surstylus. Scale = 0.1 mm.

***Medetera aglaops*** Pollet sp. nov. (Figure 1A–C, Figure 2, Figure 3, Figure 4 and Figure 5)

urn:lsid:zoobank.org:act:B1094DCC-B379-438B-B832-8D626613FA34

**Diagnosis**. Small species, wing length 1.9–2.4 mm. Face with epistoma and clypeus strongly dusted greyish, and epistoma with multiple shallow diverging furrows. Eye with red base colour and brilliant green reflection in the lower 2/3, and dark violet in about the upper 1/3 (with smaller ommatidia). Mesonotum (incl. scutellum) bluish grey, strongly dusted, with rusty brown area between dc rows. Five dc with first dc small, about 0.4× as long as second dc bristle; more than six microscopic ac; one spal bristle. Wing slightly smoked brownish; halter yellowish white. All legs overall blackish brown, femora I–III with narrow reddish yellow knees. Tibia II with ad and pd bristle pair. Surstylus rather slender and S-shaped, with distinct split in ventral and dorsal lobe at half length; hypandrium straight dorsally (lateral view), rocket-shaped with lateral hooks at basal 1/3, and two dark stripes at apex (ventral view).

**Description**. **Male**. Body length: 1.9–2.4 mm; wing length: 2.0–2.3 mm, wing 0.4× as wide as long. **Head**. Face with both epistoma and clypeus strongly dusted greyish; epistoma sometimes with less dusted bronze spot in centre, narrowing below antennae, parallel-sided in middle (1/3 of eye width), and slightly diverging at clypeus, with multiple shallow diverging furrows; clypeus about as long as wide, bare. Frons metallic dark violet blue, with strong greyish dusting. Occiput bronze, strongly dusted greyish, distinctly concave. Palpus small, 1/7 of eye height, ovoid, dark brown, dusted grey, with slight bluish reflection, with yellow pubescence and yellow ap bristle. Proboscis dark brown. Eye with red base colour and brilliant green reflection in the lower 2/3, and dark violet in about the upper 1/3 (with smaller ommatidia), bare. All postocular bristles, most rather long, gently curved, white, with uppermost three distinctly shorter. One pair of minute brown postocellar bristles. Antenna entirely black, with scape bare and pedicel with apical crown of small bristles; postpedicel subcircular, rarely with small blunt apv process, about as long as deep, and about 0.8× as long as scape and pedicel combined, with microscopic pubescence; stylus apical, about 3.3× as long as first three antennal segments combined, with very short first segment, bare. **Thorax**. Mesonotum (incl. scutellum) bluish grey, strongly dusted, with rusty brown area between dc rows; pleura metallic bronze to bluish green, dusted greyish. Thoracic bristles black. Five dc (two presutural, one sutural, two postsutural), with first dc small, about 0.4× as long as second dc, and dc2–5 strong. More than six ac, biseriate, reaching beyond third dc, microscopic; three (one large black, two minute white) ant pprn, one external bas pprn, one rather strong sut ial, one npl, one spal, and one pal bristles. Scutellum with four bristles, lateral ones rather small, about 0.5× as long as median ones. With 2–3 white prothoracic bristles of subequal length, and about 3–5 minute pale setae between ant pprn bristle and first dc. **Wing**. Slightly smoked brownish, with pale brown veins. Vein R_4+5_ smoothly sinuous, M_1_ straight, both distinctly converging towards wing apex; distance at wing apex 0.5× distance at crossvein dm-cu. Proximal section of M_1_ 0.9× as long as apical section. Proximal section of CuA_1_ 3.2× as long as apical section. CuA_x_ ratio: 0.8. Halter yellowish white, calypteral fringe yellow. **Legs**. Overall blackish brown, femora I–III with narrow reddish yellow knees, especially femur II. With most bristles pale, only ad and pd bristles on tibia II brown. Coxa I blackish brown, with whitish yellow pubescence nearly exclusively on anterior face (lateral face nearly entirely bare), and four white ap bristles. Coxa II dark brown, with pubescence only on anterior face, and three white bristles on anterolateral margin, with the strongest bristle most apical. Coxa III blackish brown, with white external bristle, inserted at about basal 2/5 in anterior 1/3. Trochanters blackish brown, trochanter II with three minute white anterior bristles. Femur I blackish brown, with extreme apex paler. Femora II–III blackish brown, with apex reddish yellow, femur II with short white av and vt setae (longer than in *Medetera lusitana* sp. nov.). Femur III with one vt row of short white setae on apical 1/2, and with inconspicuous basodorsal row of small white bristles on basal 1/3, about 0.3× as long as femur is deep, with basal five bristles more erect. Tibiae I–III blackish brown. Tibia I without distinct bristles. Tibia II with one ad bristle at less than basal 1/3, and one pd bristle at basal 1/3, former not as long as tibia is deep and latter about as long as tibia is deep; with four small ap bristles. Tibia III with apicodorsal notch and weak ds serration, including one strongly inclined white bristle at apical 1/5, about 1.5× as long as tibia is deep, and two indistinct av bristles. Tarsi I–III blackish brown. Tarsus I: taI_1_ with minute yellow ventral setae, and taI_1–4_ with apv crown of dark setae. Tarsus II: taII_1–5_ with many minute yellow and some darker setae, and taII_1–4_ with apv crown of dark setae. Tarsus III: taIII_2–4_ with apv crown of dark setae, and taIII_1_ with indistinct blunt pv tooth at basis (MSSC). Ratio of femur/tibia/tarsomeres 1–5 in leg I: 10/9.3/4.4/2.1/1.6/1/1.3, in leg II: 8.9/9.2/5.1/2.5/1.7/0.9/1, and in leg III: 8.5/10.4/2.6/3.3/2/1/1. **Abdomen**. With seven pubescent segments. Tergites greenish to bluish bronze, brilliant on dorsum, more dusted on sides, with white pubescence and short bristles on posterior margins; bristles on sides of T_1_ erect and large. Sternites dark brown, with weak metallic green reflection and weak dusting, with white, very short pubescence; S_2–5_ with blunt incision in middle of posterior margin, S_6_ entirely unsclerotised. Hypopygium incl. epandrium brownish black; hypandrium reddish brown, rocket-shaped, with lateral hooks at basal 1/3 and two dark stripes at apex; with one small basal, and two larger more apical epandrial setae, latter on rather long stalks; surstylus dark brown, rather slender and S-shaped, with clear split in vt and ds lobe at less than apical ½; vt lobe with one modified seta; cercus pale brown, rather small, elongate triangular, with two ap flattened bristles.

**Female**. As male, except for: body length: 2.4–2.9 mm; wing length: 2.1–2.7 mm. Face 2/5 of eye width in middle. Wing with proximal section of CuA_1_ 3.5× as long as apical section; CuA_x_ ratio: 0.9. Abdomen with five pubescent segments; segments 6–11 telescopic, and 12th segment with two black needle-like acanthophorites and two equal-sized brown cerci. Sternites coloured as in male but S_2–5_ complete. Tarsomere III_1_ without blunt basal pv tooth. Ratio of femur/tibia/tarsomeres 1–5 in leg I: 10.3/9.6/4.6/2.3/1.6/1/1.3, in leg II: 8.7/8.9/4.9/2.4/1.6/0.9/1, and in leg III: 8.2/10.8/2.6/3.5/2/1/1.

**Type material. HOLOTYPE**: 1♂, **SPAIN**: Aragón, Huesca, Fraga (very dry habitat mostly covered by sclerophyllous shrubs and patches of pine forest) (Ma_10), 41°30′40.7″ N, 0°16′32.8″ E, 340 m, 14.v.2015, SW, leg. Rui Andrade—sample cd: ES/2015/259/RA [RBINS, IG: 34520/001].

**PARATYPES**: **SPAIN**: 1♀, Andalucía, Almería, Abrucena (rivulet in the mountains) (Ma_4), 37.1134, −2.8363, 1215 m, 6.v.2015, SW, leg. Francisco Rodríguez Luque—sample cd: ES/2015/006d/PA; 1♂, Laujar de Andarax (on rocks) (Ma_5), 37.09063, −2.91408, 2343 m, 12.v.2017, SW, leg. Francisco Rodríguez Luque—sample cd: ES/2017/003/PA; 1♀, Vícar (stony ground) (Ma_6), 36.8389, −3.78336, 342 m, 27.iii.2015, SW, leg. Francisco Rodríguez Luque—sample cd: ES/2015/002a/PA; 3♀, Sevilla, Corredor Verde (Ma_3), 37.3968, −6.2285, 12.iii.2020, SW, leg. J. Mortelmans and D. Volckaert—sample cd: ES/2020/004/JM; 1♂, 1♀, Granada, Acequias (dry river bed) (Ma_7), 36.9629, −3.54654, 780 m, 24.iv.2015, SW, leg. Piluca Álvarez—sample cd: ES/2015/006c/PA; 4♂, El Cerro Gordo (on rocks in stony slope) (Ma_8), 36.73355, −3.7711, 58 m, 25.iv.2015, SW, leg. Piluca Álvarez Fidalgo—sample cd: ES/2015/007b/PA; 3♂, 1♀, Lobres (on rocks in dry riverbed) (Ma_9), 36.7860, −3.5327, 97 m, 25.iv.2015, SW, leg. Piluca Álvarez Fidalgo—sample cd: ES/2015/001b/PA; 9♂, 3♀, same data as holotype; 1♂, Castilla-La Mancha, Toledo, Argés, Guajaraz Reservoir (on rocks by reservoir) (Ma_11), 39.79655, −4.08379, 605 m, 26.iv.2017, SW, leg. Piluca Álvarez Fidalgo—sample cd: ES/2017/001/PA [MNCN]; 2♂, 1♀, Castilla y León, Burgos, Villaquirán de los Infantes (wall of building in agricultural landscape) (Ma_12), 42°12′59.6″ N, 4°0′08.1″ W, 780 m, 11.v.2015, HC, leg. Ana Gonçalves and Rui Andrade—sample cd: ES/2015/231/AG&RA; 1♀, Burgos, Villasur de Herreros (shrubland) (Ma_13), 42°18′42.2″ N, 3°23′34.0″ W, 1030 m, 15.v.2014, SW, leg. Rui Andrade—sample cd: ES/2014/215/RA; 1♂, Cataluña, Lleida, Pallars Sobirà, Sort (Llessui) (mountainous mosaic area of pastures and forests) (Ma_14), 42°27′03.6″ N, 1°04′14.6″ E, 1410 m, 4.viii.2014, SW, leg. Rui Andrade and Ana Gonçalves—sample cd: ES/2014/216/RA&AG; 1♀, Comunidad de Madrid, Madrid, Aranjuez (rest area along road) (Ma_15), 42°12′12.2″ N, 0°12′44.6″ E, 550 m, 14.v.2015, HC, leg. Rui Andrade—sample cd: ES/2015/315/RA; 1♂, Extremadura, Badajoz, Valencia del Ventoso (small river crossing Mediterranean shrubland) (Ma_16), 38°16′54.4″ N, 6°33′13.3″ W, 325 m, 2.iv.2015, SW, leg. Rui Andrade—sample cd: ES/2015/175/RA; 1♂, ES: Extremadura, Cáceres, Abertura (Mediterranean forest and shrubland) (Ma_17), 39°34′24.0″ N, 4°47′17.0″ W, 790 m, 15.iv.2016, SW, leg. Rui Andrade and Ana Gonçalves—sample cd: ES/2016/337/RA&AG; 1♀, Galicia, Lugo, San Martiño (grassy river bank) (Ma_1), 42°23′26.5″ N, 7°11′3.5″ W, 300 m, 12.x.2007, SW, leg. J.L. Camaño Portela—sample cd: ABIGA/8302; 1♀, Ourense, Rubiá, Covas (mountainous forested site) (Ma_18), 42°28′56.2″ N, 6°50′13.0″ W, 470 m, 11.v.2014, SW, leg. Rui Andrade—sample cd: ES/2014/236/RA; 1♂, Lugo, Folgoso do Courel, Teixeira (open field on limestone soil) (Ma_2), 42°41′24.4″ N, 7°9′38.9″ W, 1250 m, 14.vi.2009, SW, leg. J.L. Camaño Portela—sample cd: ABIGA/64729; 1♂, Valencia, Alicante, Alfaz del Pi (entrance of cave) (Ma_19), 38°34′45.1″ N, 0°06′04.4″ W, 4.iv.2008, YPT/RPT, leg. A. Sendra—sample cd: ES/250308ST82/A006; 1♂, 2♀, same data—sample cd: ES/250308ST58/A005; 1♂, 1♀, same data—sample cd: ES/250308ST96/A007; 1♂, Valencia, Terrenos de labor, Enguera (almond tree plantation) (Ma_20), 38°58′42.6″ N, 0°41′20.9″ W, 6.v.2006, YPT/RPT, leg. Sergio Montagud et al.—sample cd: ES/290508PJ51. **FRANCE**: 1♀, Corse-du-Sud (2A), Quenza, Plateau du Coscione, Castellu d’Ornucciu (mountain alder forest) (Ma_21), 41.8327255249023, 9.15901279449463, 1636 m, 26–30.vi.2019, YPT, leg. Claire Villemant—sample cd: FR-COR/2020/025/MNHN; 1♂, 2♀, Sorbollano, Campu di Bonza (maquis landscape) (Ma_22), 41.7698669433594, 9.12492656707764, 890 m, 22.ii–6.vi.2020, MT, leg. Eddy Poirier, Rémy Poncet and Julien Touroult—sample cd: FR-COR/2020/147/MNHN; 1♂, Zicavo (Ma_23), 41.876356, 9.132745, 1244 m, 25–29.vi.2019, YPT, leg. Romain Le Divelec—sample cd: FR-COR/2020/176/MNHN; 1♂, 2♀, Haute-Corse, Oletta (Ma_24), 42.65401178095543, 9.295872382020844, 18–21.x.2020, RsPT, leg. Romain Le Divelec—sample cd: FR-COR/2020/086/MNHN; 2♂, 4♀, Palasca (Ma_25), 42.6639556884766, 9.06591701507568, 87 m, 13–22.x.2020, leg. Jean Ichter, Eddy Poirier, Julien Touroult and Camille Gazay—sample cd: FR-COR/2020/060/MNHN; 3♂, 3♀, Palasca, Vadellare stream, Gradu (riparian forest) (Ma_26), 42.659481048584, 9.07185173034668, 5 m, 13–22.x.2020, MT, leg. Eddy Poirier—sample cd: FR-COR/2020/062/MNHN; 1♂, 1♀, Corse-du-Sud (2A), Serra di Scopamène, Campu di Bonza (Ma_27), 41°46′20.3226″ N, 9°7′22.4364″ E, 980 m, 14.vi.2019–27.vi.2019, MT, leg. Julien Touroult—sample cd: FR-COR/2019/220; 1♂, 2♀, Serra di Scopamène, Castellu d’Ornucciu (in higher mountain alder forest) (Ma_28), 41°49′58.6″ N, 09°09′26.1″ E, 1580 m, 26–30.vi.2019, BPT, leg. Marc Pollet—sample cd: FR-COR/2019/146; 1♀, same site, WPT, leg. Marc Pollet—sample cd: FR-COR/2019/145; 6♂, 2♀, same location (in lower mountain alder forest) (Ma_29), 41°49′59.6″ N, 09°09′26.4″ E, 1556 m, 26–30.vi.2019, BPT, leg. Marc Pollet—sample cd: FR-COR/2019/149; 2♂, same site, WPT, leg. Marc Pollet—sample cd: FR-COR/2019/148; 1♀, same site, YPT, leg. Marc Pollet—sample cd: FR-COR/2019/147; 7♂, 4♀, same location (on rocks at edge of forest and pozzine habitats) (Ma_30), 41°49′59.9″ N, 09°09′29.2″ E, 1571 m, 26.vi.2019, HC, leg. Marc Pollet—sample cd: FR-COR/2019/058; 2♂, 2♀, same location (in shady sites along stream in pozzine landscape) (Ma_31), 41°50′00.5″ N, 09°09′27.6″ E, 1568 m, 26–30.vi.2019, WPT, leg. Marc Pollet—sample cd: FR-COR/2019/151; 1♂, 3♀, same location (in open rocky sites along stream in pozzine landscape) (Ma_32), 41°50′02.9″ N, 09°09′24.2″ E, 1559 m, 26.vi.2019–30.vi.2019, BPT, leg. Marc Pollet—sample cd: FR-COR/2019/156; 1♀, Serra di Scopamène et Sorbollano, Campu di Bonza (on gravelly muddy seep in deciduous forest) (Ma_33), 41°46′21.4″ N, 09°07′16.2″ E, 935 m, 23–27.vi.2019, BPT, leg. Marc Pollet—sample cd: FR-COR/2019/085; 2♂, same location (in brook bed in oak forest) (Ma_34), 41°46′21.9″ N, 09°07′15.1″ E, 934 m, 23–27.vi.2019, BPT, leg. Marc Pollet—sample cd: FR-COR/2019/091; 1♀, same location (on banks of river in oak forest) (Ma_35), 41°46′28.3″ N, 09°07′26.9″ E, 845 m, 23–27.vi.2019, BPT, leg. Marc Pollet—sample cd: FR-COR/2019/074; 4♀, same location (Ma_22), 41°46′11.5206″ N, 9°7′29.7366″ E, 936 m, 14–27.vi.2019, MT, leg. Julien Touroult—sample cd: FR-COR/2019/211; 1♀, same site, 27.vi–11.vii.2019, MT, leg. Julien Touroult—sample cd: FR-COR/2019/213; 2♀, same site, 19.ix–3.x.2019, MT, leg. Julien Touroult—sample cd: FR-COR/2019/209; 3♂, 2♀, Sorbollano, Campu di Bonza (Ma_36), 41°46′11.823″ N, 9°7′30.1692″ E, 929 m, 14–27.vi.2019, MT, leg. Julien Touroult—sample cd: FR-COR/2019/233; 1♂, 3♀, same location (Ma_37), 41°46′15.8046″ N, 9°7′27.606″ E, 949 m, 14.vi–27.vi.2019, MT, leg. Julien Touroult—sample cd: FR-COR/2019/212; 1♂, 1♀, same site, 27.vi–11.vii.2019, MT, leg. Julien Touroult—sample cd: FR-COR/2019/219; 1♀, Zicavo, Ponte di Valpine (on rocks in grassy habitat nr river) (Ma_38), 41°52′33.0″ N, 09°08′00.2″ E, 1251 m, 25.vi.2019, HC, leg. Marc Pollet—sample cd: FR-COR/2019/051; 1♀, Zonza, Samulaghia (on rocks at small pool/spring along the trail) (Ma_39), 41°45.667′ N, 09°13.544′ E, 1154 m, 24.vi.2019, SW, leg. Marc Pollet—sample cd: FR-COR/2019/030; 6♂, 3♀, same location (in dry fir (*Abies* sp.) forest) (Ma_40), 41°45.697′ N, 09°13.658′ E, 1209 m, 24–28.vi.2019, BPT, leg. Marc Pollet—sample cd: FR-COR/2019/098; 1♀, same site, 24–28.vi.2019, WPT, leg. Marc Pollet—sample cd: FR-COR/2019/097; 1♀, same site, 24–28.vi.2019, YPT, leg. Marc Pollet—sample cd: FR-COR/2019/096; 10♂, 15♀, Zonza, Samulaghia (on dry rocks near seep in fir forest) (Ma_41), 41°45.703′ N, 09°13.649′ E, 1208 m, 24–28.vi.2019, BPT, leg. Marc Pollet—sample cd: FR-COR/2019/101; 1♂, same site, 28.vi.2019, HC, leg. Anja De Braekeleer—sample cd: FR-COR/2019/118; 4♂, 1♀, same site, 24–28.vi.2019, WPT, leg. Marc Pollet—sample cd: FR-COR/2019/100; 10♂, 6♀, same location (marshy seep in dry fir forest) (Ma_42), 41°45′39.6″ N, 9°13′37.2″ E, 1244 m, 24–28.vi.2019, BPT, leg. Marc Pollet—sample cd: FR-COR/2019/109; 1♀, same site, 24–28.vi.2019, WPT, leg. Marc Pollet—sample cd: FR-COR/2019/108; 4♂, same site, 24–28.vi.2019, WPT, leg. Marc Pollet—sample cd: FR-COR/2019/110; 1♀, Zonza, Samulaghia (seep on rocks in fir forest) (Ma_43), 41°45′40.1″ N, 09°13′32.9″ E, 1188 m, 28.vi.2019, HC, leg. Marc Pollet—sample cd: FR-COR/2019/115; 2♂, 3♀, same location (on rocky seep in fir forest (edge of forest)) (Ma_44), 41°45′40.1″ N, 9°13′32.9″ E, 1231 m, 24–28.vi.2019, BPT, leg. Marc Pollet—sample cd: FR-COR/2019/114; 3♂, 2♀, same location (at little seep along trail) (Ma_45), 41°46.022′ N, 09°13.409′ E, 1117 m, 24.vi.2019, HC, leg. Marc Pollet—sample cd: FR-COR/2019/031; 1♀, same location (canopied seep along the road at edge of forest) (Ma_46), 41°46.119′ N, 09°13.347′ E, 1120 m, 24.vi.2019, PT, leg. Marc Pollet—sample cd: FR-COR/2019/033; 2♂, same location (on dry rocky and concrete construction on stream) (Ma_47), 41°46.127′ N, 09°13.375′ E, 1095 m, 24.vi.2019, SW, leg. Marc Pollet—sample cd: FR-COR/2019/024; 2♂, 1♀, same location (on concrete bridge) (Ma_48), 41°46.129′ N, 09°13.380′ E, 1115 m, 24.vi.2019, HC, leg. Marc Pollet—sample cd: FR-COR/2019/034; 1♀, same location (on rocks at small waterfall on stream) (Ma_49), 41°46.133′ N, 09°13.378′ E, 1116 m, 24.vi.2019, SW, leg. Marc Pollet—sample cd: FR-COR/2019/032, all as part of the La Planète Revisitée Corsica MNHN-PNI 2019–2021 expedition; 2♂, 1♀, Provence-Alpes-Côte d’Azur, Alpes-Maritimes, Mandelieu, Esterel (on large stones in maquis landscape) (Ma_50), 43.52177, 6.89584, 70 m, 4.v.2022, HC, leg. Frédéric Belin—sample cd: FR/2022/045/FB. **ITALY**: 1♂, Sardinia, Carbonia-Iglesias, Domusnovas, Valle Oridda (on left bank of rio d’Oridda on plateau with secondary garrigue) (Ma_51), 39.408932° N, 8.616372° E, 592 m, 18.iv–2.v.2006, MT, leg. G. Chessa—sample cd: IT/2006/009/GN (Progetto Sardegna—CNBF 2006); 3♂, 2♀, Iglesias, Marganai, Tintillonis (clearing in forest) (Ma_52), 39.345887° N, 8.570726° E, 480 m, 22–25.ix.2004, MT, leg. D. Birtele, P. Cerretti, E. Gatti, F. Mason and D. Whitmore—sample cd: IT/2006/024/GN (Conecofor Programme—CNBF 2003); 2♂, Iglesias, nr colonia Beneck (in shrubby vegetation at edge of *Quercus suber* forest in valley) (Ma_53), 39.347590° N, 8.563532° E, 636 m, 18.iv–2.v.2006, MT, leg. G. Chessa—sample cd: IT/2006/001/GN (Progetto Sardegna—CNBF 2006); 1♂, same site, 2–16.v.2006, MT, leg. G. Chessa—sample cd: IT/2006/015/GN (Progetto Sardegna—CNBF 2006); 1♂, 1♀, same site, 30.v–13.vi.2006, MT, leg. G. Chessa—sample cd: IT/2006/029/GN (Progetto Sardegna—CNBF 2006). **PORTUGAL**: 5♂, 1♀, Alto Alentejo, Santa Maria de Marvão (Mediterranean shrubland with big rocks) (Ma_54), 39°23′49.2″ N, 7°21′51.4″ W, 610 m, 14.iii.2015, SW, leg. Rui Andrade—sample cd: PT/2015/183/RA; 1♂, Beira Alta, Aldeias (mountainous site with mixed forest) (Ma_55), 40°28′05.4″ N, 7°35′15.7″ W, 855 m, 25.iv.2013, SW, leg. Rui Andrade—sample cd: PT/2013/082/RA; 1♀, Loriga, Praia Fluvial de Loriga (rocky mountain river) (Ma_56), 40°19′41.26″ N, 7°40′36.71″ W, 890 m, 3.ix.2014, SW, leg. Ana Rita Gonçalves—sample cd: PT/2014/204/AG; 1♂, Sabugueiro, Lagoa Comprida (dam in rocky mountain area) (Ma_57), 40°21′54.3″ N, 7°38′32.5″ W, 1600 m, 21.ix.2013, SW, leg. Rui Andrade—sample cd: PT/2013/227/RA; 1♀, Beira Litoral, Sé Nova, Jardin Botânica de Coimbra (botanical garden; most specimens were collected near ponds or on walls) (Ma_58), 40°12′20.0″ N, 8°25′15.0″ W, 85 m, 9.v.2013, HC, leg. Ana Rita Gonçalves—sample cd: PT/2013/110/AG; 1♂, Vidual (dam surrounded by pine forest and Mediterranean shrubland) (Ma_59), 40°05′27.5″ N, 7°51′33.6″ W, 666 m, 8.x.2015, SW, leg. Rui Andrade—sample cd: PT/2015/381/RA; 1♂, 3♀, Douro Litoral, Oliveira do Douro, Parque Municipal da Lavandeira (extensive lawns and a small stream bordered by trees) (Ma_60), 41°07′18.4″ N, 8°35′40.9″ W, 100 m, 20.v.2009, HC, leg. Rui Andrade—sample cd: PT/2009/135/RA; 1♂, Valongo, Serras de Valongo (mountainous site crossed by a small river) (Ma_61), 41°09′33.4″ N, 8°29′05.6″ W, 75 m, 6.v.2010, SW, leg. Rui Andrade—sample cd: PT/2010/001/RA; 3♂, Valongo, Serras de Valongo (mountainous site crossed by a small river) (Ma_62), 41°09′33.4″ N, 8°29′05.6″ W, 75 m, 17.v.2010, SW, leg. Rui Andrade—sample cd: PT/2010/006/RA; 1♀, same site, 13.vi.2012, SW, leg. Rui Andrade—sample cd: PT/2012/135/RA; 1♂, same site, 23.v.2013, SW, leg. Rui Andrade—sample cd: PT/2013/034/RA; 1♀, Estremadura, A dos Cunhados, Casal da Serra (agricultural fields) (Ma_63), 39°09′56.20″ N, 9°20′31.53″ W, 50 m, 19.ix.2013, HC, leg. Rui Andrade—sample cd: PT/2013/292/RA; 1♂, 2♀, same site, 22.viii.2013, SW, leg. Rui Andrade—sample cd: PT/2013/237/RA; 6♂, 5♀, same site, 19.ix.2013, SW, leg. Rui Andrade—sample cd: PT/2013/185/RA; 1♂, same site, 19.ix.2013, SW, leg. Rui Andrade—sample cd: PT/2013/185/RA; 3♂, Tornada, Paul de Tornada (marshland) (Ma_64), 39°26′52.8″ N, 9°08′04.0″ W, 10 m, 20.ix.2012, SW, leg. Rui Andrade—sample cd: PT/2012/026/RA; 2♂, 3♀, same site, 22.ix.2012, SW, leg. Rui Andrade—sample cd: PT/2012/106/RA; 1♀, same site, 26.ix.2012, SW, leg. Rui Andrade—sample cd: PT/2012/037/RA; 3♀, Minho, Apúlia, Parque Natural do Litoral Norte (Ma_65), 41°28′16.4″ N, 8°46′27.2″ W, 2 m, 23.v.2012, SW, leg. Rui Andrade—sample cd: PT/2012/069/RA; 1♂, same site, 23.v.2012, SW, leg. Rui Andrade—sample cd: PT/2012/069/RA; 1♂, same site, 19.iv.2015, SW, leg. Rui Andrade—sample cd: PT/2015/323/RA; 1♀, Gilmonde (agricultural fields surrounded by small patches of forest) (Ma_66), 41°30′42.0″ N, 8°38′56.1″ W, 37.5 m, 21.v–4.vi.2012, MT, leg. Rui Andrade—sample cd: PT/2012/325/RA; 9♂, 5♀, same location, 41°30′43.0″ N, 8°38′57.0″ W (Ma_67), 37.5 m, 8.v.2009, HC, leg. Rui Andrade—sample cd: PT/2009/137/RA; 4♂, 1♀, same site, 21.v.2012, SW, leg. Rui Andrade—sample cd: PT/2012/117/RA; 2♂, 3♀, same site, 4.vi.2012, SW, leg. Rui Andrade—sample cd: PT/2012/015/RA; 2♂, 2♀, same site, 14.iv.2013, SW, leg. Rui Andrade—sample cd: PT/2013/007/RA; 1♂, Rio Tinto, Marachão (mosaic landscape on banks of the Cávado river) (Ma_68), 41°30′16.9″ N, 8°43′10.6″ W, 23 m, 15.iv.2013, SW, leg. Rui Andrade—sample cd: PT/2013/020/RA; 1♀, Vilar da Veiga, Gerês (woodland in a mountainous area) (Ma_69), 41°44′16.7″ N, 8°09′19.8″ W, 500 m, 24.vii.2010, HC, leg. Rui Andrade—sample cd: PT/2011/235/RA; 1♀, Ribatejo, Olaia, Lamarosa (Ma_70), 39°31′46.66″ N, 8°28′10.13″ W, 65 m, 30.iv.2013, SW, leg. Ana Rita Gonçalves—sample cd: PT/2013/154/AG; 1♂, Santa Margarida da Coutada (riparian corridor of a small stream) (Ma_71), 39°26′23.3″ N, 8°16′50.7″ W, 75 m, 30.iii.2011, SW, leg. Rui Andrade—sample cd: PT/2011/004/RA; 1♂, Trás-os-Montes e Alto Douro, Adeganha, Estevais (Mediterranean sclerophyllous forest and shrubland) (Ma_72), 41°13′58.8″ N, 7°04′22.0″ W, 449 m, 1.vi.2016, SW, leg. Rui Andrade—sample cd: PT/2016/362/RA; 2♀, Algoso (riparian corridor crossing Mediterranean shrubland) (Ma_73), 41°27′17.6″ N, 6°35′35.2″ W, 341 m, 16.vi.2015, SW, leg. Rui Andrade—sample cd: PT/2015/310/RA; 1♂, Algoso, Vale de Algoso (small plots of agricultural land, surrounded by Mediterranean shrubland) (Ma_74), 41°29′52.4″ N, 6°32′19.9″ W, 575 m, 5.x.2015, SW, leg. Rui Andrade and Ana Gonçalves—sample cd: PT/2015/379/RA&AG; 1♂, 1♀, same site, 6.x.2015, SW, leg. Rui Andrade and Ana Gonçalves—sample cd: PT/2015/348/RA&AG; 1♂, Castrelos (riparian corridor in a mountainous area) (Ma_75), 41°50′22.1″ N, 6°53′24.2″ W, 611 m, 2.x.2015, SW, leg. Rui Andrade—sample cd: PT/2015/350/RA; 1♀, Ermelo, Varzigueto (mountain river crossing meadows) (Ma_76), 41°22′44.0″ N, 7°51′14.4″ W, 750 m, 25.vi.2013, SW, leg. Rui Andrade—sample cd: PT/2013/008/RA; 1♂, Gondesende (riparian corridor in mountainous area crossing meadow and *Quercus pyrenaica* forest) (Ma_77), 41°50′47.1″ N, 6°52′45.9″ W, 638 m, 10.v.2015, SW, leg. Rui Andrade—sample cd: PT/2015/264/RA; 1♂, Meirinhos (lowland river in ravine) (Ma_78), 41°17′13.4″ N, 6°52′21.5″ W, 222 m, 17.vi.2015, SW, leg. Rui Andrade—sample cd: PT/2015/321/RA; 2♂, 1♀, Pitões das Júnias (oak forest with small waterfall) (Ma_79), 41°49′48.7″ N, 7°57′01.3″ W, 965 m, 10.ix.2012, SW, leg. Rui Andrade—sample cd: PT/2012/051/RA; 1♀, Silva, Santo Adrião (ponds as remnants of drying up river amid Mediterranean forest) (Ma_80), 41°31′48.2″ N, 6°28′27.24″ W, 552 m, 1.iv.2015, SW, leg. Ana Rita Gonçalves—sample cd: PT/2015/404/AG; 2♂, Vila Chã de Braciosa (lowland river in ravine) (Ma_81), 41°25′29.8″ N, 6°18′29.4″ W, 603 m, 6.x.2015, SW, leg. Rui Andrade—sample cd: PT/2015/344/RA; 1♂, same site, 3.vi.2016, SW, leg. Rui Andrade—sample cd: PT/2016/366/RA; 2♂, 1♀, Vinhais (forest of *Quercus pyrenaica* and conifers)(Ma_82), 41°51′01.3″ N, 6°59′21.6″ W, 983 m, 10.ix.2014, SW, leg. Rui Andrade—sample cd: PT/2014/225/RA [RBINS, IG: 34520/002; and MAPC, unless otherwise mentioned].

**Distribution**. Portugal (Alto Alentejo, Beira Alta, Beira Litoral, Douro Litoral, Estremadura, Minho, Ribatejo, Trás-os-Montes e Alto Douro), Spain (Andalucía, Aragón, Castilla-La Mancha, Castilla y León, Cataluña, Comunidad de Madrid, Extremadura, Galicia, Comunidad Valenciana), France (Provence-Alpes-Côte d’Azur, Corsica) and Italy (Sardinia).

**Etymology**. The specific epithet “*aglaops*” is retrieved from the ancient Greek Ἀγλαωψ, which means “with beautiful eyes”, referring to the unusual colour pattern of the eyes in frontal and lateral view.

**Ecology**. *M. aglaops* sp. nov. has been encountered both in natural biotopes and agricultural landscapes from 2 m up to 2343 m a.s.l. It is mostly found in rather dry sites, on rocks or other hard substrates, often in forests but also in more open habitats. It has been collected in 83 different sites and four countries and as such is the most abundant and widespread of all new *Medetera* species described here. It was encountered in five samples together with *M. lusitana* sp. nov., and in four other samples together with one of the following species: *M. corsicana* sp. nov., *M. gibbosipyga* sp. nov., *M. hispanica* sp. nov., *M. rectipyga* sp. nov. During the Our Planet Reviewed expedition in Corsica (2019), *M. aglaops* sp. nov. was collected in highest numbers along a seep on rocky substrate in a fir (*Abies* sp.) forest above 1200 m a.s.l. In this site, yields were far higher in blue pan traps (*n* = 55) than in white (*n* = 11) or yellow traps (*n* = 1). This is a phenomenon that has been observed previously in tree-trunk dwelling species, and *Medetera* in particular e.g., [29,30].

**Figure 6:**
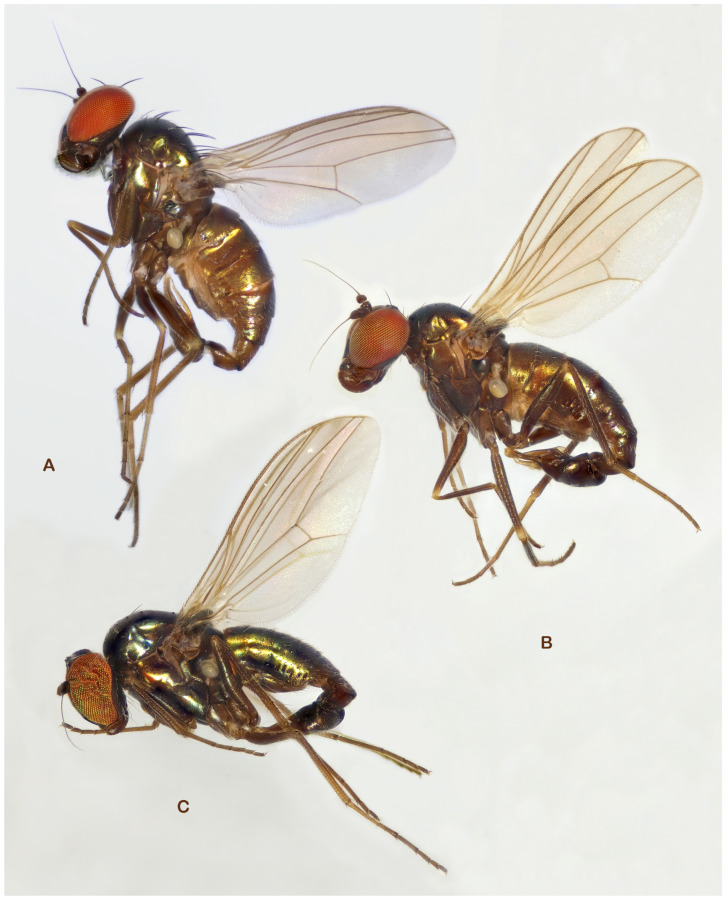
*Medetera* species, general habitus (**A**) *Medetera corsicana* sp. nov.; (**B**) *Medetera gibbosipyga* sp. nov.; (**C**) *Medetera hispanica* sp. nov. (photos: Andreas Stark).

**Figure 7:**
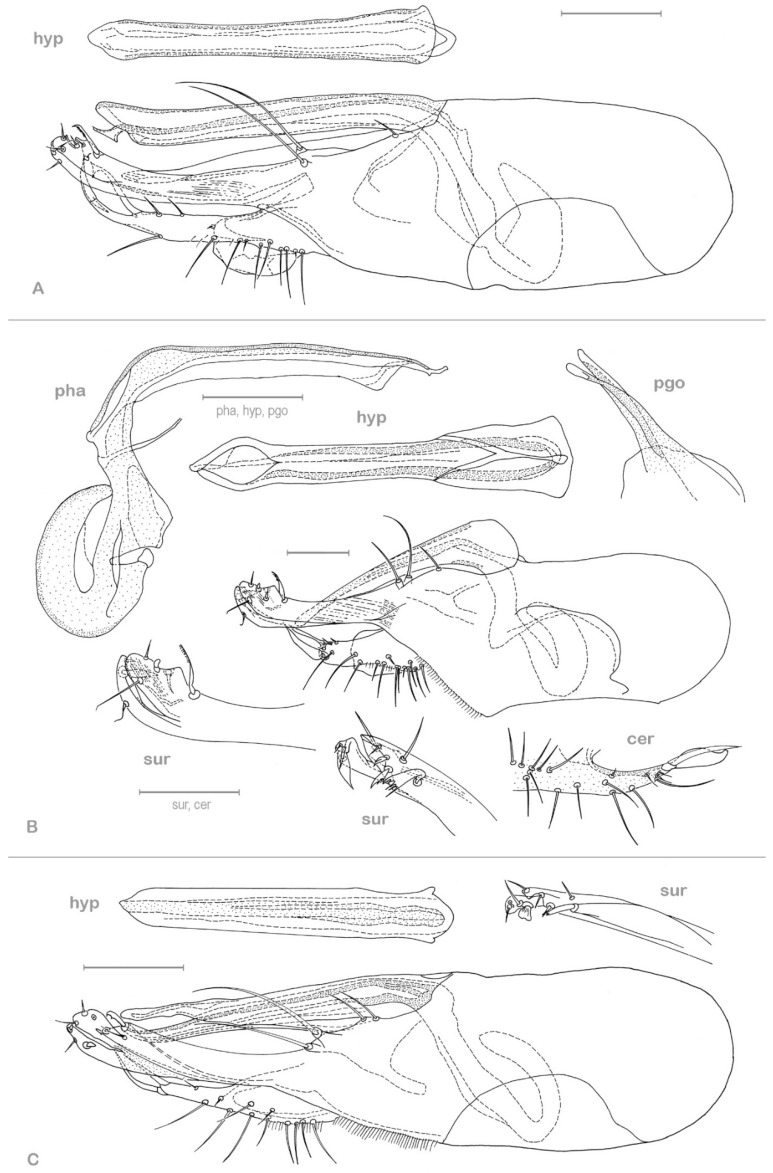
*Medetera* hypopygia, lateral view (**A**) *Medetera corsicana* sp. nov.; (**B**) *Medetera gibbosipyga* sp. nov.; (**C**) *Medetera hispanica* sp. nov. Cer: cercus, hyp: hypandrium, pha: phallus, pgo: postgonite, sur: apical part of surstylus. Scale = 0.1 mm.

***Medetera corsicana*** Pollet sp. nov. (Figure 2, Figure 6A and Figure 7A)

urn:lsid:zoobank.org:act:5F10B2FF-2B2A-4ACA-A777-480F1DE66FCE

**Diagnosis**. Very small species, wing length 1.7–1.9 mm (*n* = 3). Face with epistoma green, heavily dusted whitish; clypeus brilliantly green in middle. Eye uniformly red. Mesonotum uniformly metallic green–bronze. Five dc; four ac. Wing very slightly infuscate; halter with yellow knob and infuscate shaft. Coxae and femora overall blackish brown, with pale knees; tibiae brownish to reddish yellow, and tarsi with tarsomeres 1–2 pale with dark apex, and tarsomeres 3–5 dark. Tibia II with ad and pd bristle pair. Surstylus with indistinct apical split; hypandrium rather straight with weak bend at apex (lateral view), slightly tapering towards triangular apex (ventral view).

**Description**. **Male**. Body length: 1.9–2.1 mm (*n* = 3); wing length: 1.7–1.9 mm (*n* = 3), 0.2× as wide as long. **Head**. Face with epistoma green, heavily dusted whitish, and clypeus brilliantly green in middle, narrowing below antennae, parallel-sided in middle (1/2 of eye width) and slightly diverging at clypeus; epistoma 2× as high as clypeus, latter slightly longer than wide, bare. Frons metallic green, with strong whitish dusting. Occiput bronze, with slight greyish dusting, distinctly concave. Palpus small, about 1/8 of eye height, ovoid, dark brown, shining, with yellow pubescence and one yellow apical bristle. Proboscis dark brown. Eye with red base colour and greenish reflection. Lowermost postocular bristles white, quite long and gently curved, and uppermost slightly darker, short, and erect. One pair of minute brown postocellar bristles. Antenna black, with scape bare and pedicel with apical crown of small bristles; postpedicel black, subcircular, sometimes with small apv process, about as long as deep, and 0.7× as long as scape and pedicel combined, with microscopic pubescence; stylus apical, about 4× as long as first three antennal segments combined, with first segment very short, bare. **Thorax**. Mesonotum incl. pleura and scutellum uniformly metallic green—bronze, with strong whitish dusting; scutellum with four bristles, lateral bristles 0.5× as long as median ones. Thoracic bristles black. Five dc (two presutural, one sutural, two postsutural), with first presutural and sutural bristle distinctly smaller than other dc, other dc increasing in length towards scutellum. About four ac, biserial, reaching nearly level of third dc (sutural), microscopic; three (one large black, two minute white) ant pprn, one external bas pprn, one small sut ial, one npl, one spal (two in HT), and one pal bristles. One–two white prothoracic bristles, with lower bristle considerably longer than upper one. Anterior surface of mesonotum with sparse white minute setae. **Wing**. Very slightly infuscate. Vein R_4+5_ smoothly sinuous, M_1_ nearly straight, both distinctly converging towards wing apex; distance at wing apex 0.5×distance at crossvein dm-cu. Proximal section of M_1_ 0.9× as long as apical section. Proximal section of CuA_1_ 3.1× as long as apical section. CuA_x_ ratio: 0.8. Halter knob yellow, slightly infuscate, with infuscate shaft; calypteral fringe white. **Legs**. Coxae and femora overall blackish brown, latter with pale knees; tibiae brownish to reddish yellow, and tarsi with tarsomeres 1–2 pale with dark apex, and tarsomeres 3–5 dark. Legs largely devoid of bristles. With pale pubescence, only larger bristles brown. All coxae blackish brown; coxa I with one row of white setae on edge between anterior and lateral face, and with four white ap bristles. Coxa II with white pubescence on anterior face, and one large white bristle on anterolateral margin. Coxa III with one white external bristle, inserted in centre. Trochanters brown, trochanter II with one–three white anterior bristles. Femora blackish brown, femora I–II with apical 1/5, and femur III with extreme apex yellow. Femur III with two small av bristles and three strongly inclined bristles in basal 1/2, about 0.5× as long as femur is deep. Tibiae and tarsi variable in colour among specimens. Tibia I from brown to brownish yellow, without distinct bristles. Tibia II pale brown to brownish yellow, with one small ad bristle at less than basal 2/5 and one small pd bristle at basal 1/3, former as long as tibia is deep and latter nearly as long as tibia is deep; with four small ap bristles. Tibia III from brown to brownish yellow, with one yellow bristle at less than ap 1/6, with weak serration of short white setae along entire length, and 1–2 indistinct ap bristles. Tarsi with tarsomeres 3–5 entirely dark. Tarsus I usually with taI_1_ yellow with dark apex, and taI_2_ yellow with apical 1/2 dark (taI_1–2_ in HT only slightly paler than taI_3–5_); taI_1_ with minute yellow ventral setae, and taI_1–4_ with apv crown of dark setae. Tarsus II with taII_1–2_ pale yellow with dark apex, taII_1–5_ with several small dark vt setae, and taII_1–4_ with apv crown of dark setae. Tarsus III with taIII_1_ pale with dark apex, gradually darker from taIII_2_ onwards; taIII_1_ with two yellow apv bristles, as long as tarsomere is deep, and with quadrate pv tooth at basis (MSSC); tarsomeres III_1–4_ with indistinct small vt bristles. Ratio of femur/tibia/tarsomeres 1–5 in leg I: 7.4/7.3/3.7/1.9/1.4/1/1.1, in leg II: 8.4/8.8/4.7/2.5/1.9/1/1, and in leg III: 7.6/9.7/2.3/3.8/2.2/1.1/1. **Abdomen**. With seven pubescent segments. Tergites greenish bronze, with strong whitish dusting, with white pubescence and short bristles on posterior margins; bristles on sides of T_1_ more erect and large. Sternites dark brown, with white, very short bristles; S_2–5_ with triangular incision in middle of posterior margin, S_6_ entirely unsclerotised. Hypopygium with epandrium brownish black; hypandrium reddish yellow, elongate, slightly narrower in middle, with rounded triangular apex; phallus with subapical cavity; with one very small basoventral, and two large apicoventral epandrial setae, latter on short stalks; surstylus brown, paler at apex, rather long, gently curved with incomplete split in ds and vt lobe, latter with modified bristles; cercus grey, rather small, elongate triangular, with two ap flattened bristles.

**Female**. Unknown.

**Type material. HOLOTYPE**: 1♂, **FRANCE**: Corsica, Corse-du-Sud (2A), Sorbollano, Campu di Bonza (Mc_1), 41°46′11.5206″ N, 9°7′29.7366″ E, 936 m, 27.vi–11.vii.2019, MT, leg. Julien Touroult—sample cd: FR-COR/2019/213 [MNHN].

**PARATYPES**: **FRANCE**: 1♂, same site as holotype, 11–25.vii.2019, MT, leg. Julien Touroult—sample cd: FR-COR/2019/235 [MNHN]; 1♂, same location (Mc_2), 41°46′14.898″ N, 9°7′26.5728″ E, 947 m, 27.vi–11.vii.2019, MT, leg. Julien Touroult—sample cd: FR-COR/2019/230 (survey: (FR-COR) La Planète Revisitée Corsica MNHN-PNI 2019-2021) [MAPC].

**Distribution**. At present only known from Corsica (Corse-du-Sud).

**Etymology**. The specific epithet “*corsicana*” refers to the island (Corsica) where the species has been discovered for the first time.

**Ecology**. The type location of this species is situated in a rather dry *Quercus ilex* forest in southern Corsica, the most common forest type on this island. *M. corsicana* sp. nov. has only been retrieved from Malaise trap samples, which suggests a primarily arboreal life history, similar to many other *Medetera* species but contrary to, e.g., *M. aglaops* sp. nov.

***Medetera gibbosipyga*** Pollet sp. nov. (Figure 2, Figure 6B and Figure 7B)

urn:lsid:zoobank.org:act:F50AB636-E291-4FAA-8813-4E4428D14391

**Diagnosis**. Small species, wing length 2.0–2.8 mm. Face with epistoma and clypeus entirely covered with strong yellowish grey pollinosity. Eye with red base colour and green reflection in lower 4/5 and reddish purple in upper 1/5. Mesonotum uniformly bronze. Five dc; 1–3 ac mostly present, sometimes absent. Wing slightly infuscate; halter brownish. Legs overall mainly blackish brown, femora I–III with narrow reddish yellow knees, tarsi I–III paler, especially taI–III_1_, with taII_1–2_ largely yellowish brown. Tibia II without ad and pd bristle pair. Surstylus rather short, slightly bending, with split at apical 1/4; hypandrium with raised basis (lateral view), hypandrium rather parallel-sided with broad basis and tulip-shaped apex (ventral view).

**Description**. **Male**. Body length: 2.1–2.5 mm; wing length: 2.0–2.8 mm, wing 0.3× as wide as long. **Head**. Face (epistoma and clypeus) entirely covered with strong yellowish grey pollinosity; narrowing below antennae, parallel-sided in middle and slightly diverging at clypeus; epistoma with one shallow central furrow, 2/5 of eye width in middle, bare. Frons (no ground colour visible) with strong yellowish grey dusting. Occiput dark brown, with slight yellowish dusting, distinctly concave. Palpus small, 1/8 of eye height, ovoid, dark brown, shining, with yellow pubescence and yellow ap bristle. Proboscis dark brown. Eye with red base colour and green reflection in lower 4/5 and reddish purple in dorsal 1/5, bare. Most postocular bristles white, with lowermost rather long, gently curved, and uppermost 2–3 shorter and mostly darker. One pair of minute brown postocellar bristles. Antenna entirely black, with scape bare and pedicel with apical crown of short bristles; postpedicel subcircular, without distinct apical process, as long as deep, and about 0.8× as long as scape and pedicel combined, with microscopic pubescence; stylus apical, 4× as long as first three antennal segments combined, with very short first segment, bare. **Thorax**. Mesonotum uniformly bronze (incl. pleura and scutellum), with strong yellowish grey dusting. Thoracic bristles black. Mostly five dc (two presutural, one sutural, two postsutural), dc1 0.5× as long as dc2–3, latter 0.5× as long as dc4–5; ac entirely absent in some specimens, but usually 1–3 ac present, biseriate, microscopic, as long as distance between rows, reaching level of third dc; three (one large black, two minute white) ant pprn, one external bas pprn, one strong sut ial, one npl, one spal, and one pal bristles. Scutellum with four bristles, lateral ones rather small, about 0.5× as long as median ones. Three white prothoracic bristles of subequal length. **Wing**. Slightly infuscate with veins yellowish at basis. Vein R_4+5_ smoothly sinuous, M_1_ gently curved, both distinctly converging towards wing apex, parallel at wing apex; distance between R_4+5_ and M_1_ at wing apex 0.6× that at crossvein dm-cu. Proximal section of M_1_ 0.9× as long as apical section. Proximal section of CuA_1_ 3.1× as long as apical section. CuA_x_ ratio: 0.8. Halter brownish, calypteral fringe white. **Legs**. Overall, mainly blackish brown, femora I–III with narrow reddish yellow knees, tarsi I–III paler, especially taI–III_1_, with tarsi II_1–2_ largely yellowish brown. Legs with pale pubescence and bristles, only apical bristles of tibia II darker. Coxa I blackish brown, with white pubescence on anterior and lateral face, and four white ap bristles. Coxa II dark brown, with white pubescence on entire anterior face, and three white bristles on anterolateral margin, with the apicalmost bristle strongest. Coxa III blackish brown, with one white external bristle in centre. Trochanters blackish brown, trochanter II with three minute white anterior bristles. Femora I–III blackish brown, femora I–II with about apical 1/5 paler and femur III with less than apical 1/5 reddish yellow; femora largely devoid of bristles. Tibiae I–III dark brown, tibia II paler towards apex. Tibia I without distinct bristles. Tibia II with four ap bristles, with apv bristle largest. Tibia III with one yellow bristle at about apical 1/6, slightly longer than tibia is deep, and two indistinct av bristles; with pd serration of short white setae, especially on apical ½. Tarsus I dark brown, with taI_1_ slightly paler; taI_1–4_ with apv crown of dark setae. Tarsus II dark brown, with taII_1–2_ yellowish brown with dark apex; taII_1–5_ with several small dark ventral setae, and taII_1–4_ with apv crown of dark setae. Tarsus III dark brown, with taIII_1_ pale brown; taIII_1–4_ with apv crown of dark setae; taIII_1_ with rather acute pv tooth at basis (MSSC). Ratio of femur/tibia/tarsomeres 1–5 in leg I: 7.5/7.3/3.7/2.1/1.4/1/1.1, in leg II: 8.8/9.5/5.4/2.8/1.9/1.1/1, and in leg III: 7.6/9.6/2.6/3.7/2.1/1.1/1. **Abdomen**. With 7 pubescent segments. Tergites greenish bronze, with rather strong yellowish grey pruinosity; with white pubescence and short bristles on posterior margins; bristles on sides of T_1_ erect and large. Sternites concolorous with T, with white, very short pubescence; S_2–5_ with blunt incision in middle of posterior margin, S_6_ entirely unsclerotised. Hypopygium incl. epandrium brownish black; hypandrium reddish brown, largely parallel-sided, with wide basis and distinctly enlarged tulip-shaped apex in ventral view; basis conspicuously raised (humpback-like) in lateral view; with one small basoventral, and two larger apicoventral epandrial setae, latter on short stalks; surstylus brown, of moderate length and width, with rather robust and slightly curved apex, latter with ds and vt process separated only at apex, both with flattened or feather-shaped setae; cercus greyish brown, rather small, elongate triangular, with two flattened ap bristles.

**Female**. As male, except for body length: 2.6–2.8 mm (*n* = 2); wing length: 2.4–2.6 mm (*n* = 2), wing 0.4× as wide as long. Face 1/3 of eye width in middle. Wing with proximal section of CuA_1_ 3.5× as long as apical section; CuA_x_ ratio: 0.9. Abdomen with five pubescent segments; segments 6–11 telescopic, and 12th segment with two black needle-like acanthophorites and two equal-sized brown cerci. Sternites coloured as in male but S_2–5_ complete. Tarsomere III_1_ without acute basal pv tooth. Ratio of femur/tibia/tarsomeres 1–5 in leg I: 8.7/8.6/4.5/2,3/1.5/1/1,2, in leg II: 9.2/9.6/5.3/2.5/1.7/1/1, and in leg III: 7.8/9.7/2.6/3.7/2/1/1.

**Type material. HOLOTYPE**: 1♂, **SPAIN**: Aragón, Huesca, Fraga (very dry habitat with sclerophyllous shrubs and patches of pine forest) (Mg_1), 41°30′40.7″ N, 0°16′32.8″ E, 340 m, 14.v.2015, SW, leg. Rui Andrade—sample cd: ES/2015/259/RA [RBINS, IG: 34520/003].

**PARATYPES**: **SPAIN**: 3♂, 2♀, same data as holotype; 2♂, Cataluña, Lleida, Alta Ribagorça, El Pont de Suert (mountainous area with shrubs, grasses, some coniferous trees and a small stream running through the rocky soil) (Mg_2), 42°21′22.4″ N, 0°50′04.4″ E, 1265 m, 5.viii.2014, SW, leg. Ana Rita Gonçalves—sample cd: ES/2014/187/AG [RBINS, IG: 34520/004: 1♂; MAPC: 2♂, 2♀].

**Distribution**. Northeastern Spain (Aragón, Cataluña).

**Etymology**. The specific epithet “*gibbosipyga*” is a concatenation of “*gibbosus*” (humped) and “*pyga*” (genital apparatus), referring to the raised basis of the hypandrium in this species which is one of its main diagnostic features.

**Ecology**. *M. gibbosipyga* sp. nov. has been encountered at only two sites between 340 m and 1265 m which can be described as dry, open biotopes, with rocky substrates, shrubs, and coniferous trees.

***Medetera hispanica*** Pollet sp. nov. (Figure 2, Figure 6C and Figure 7C)

urn:lsid:zoobank.org:act:2A3914BB-60C3-4E01-B1A3-AAA187DC2C14

**Diagnosis**. Small species, wing length 2.2 mm. Face with epistoma metallic green in centre, clypeus mainly brilliantly bronze, both dusted greyish laterally. Eye with red base colour and green reflection in more than lower 2/3, upper part violet red. Mesonotum mainly metallic green on dorsum with humeral and notopleural areas metallic violet blue. Four dc; 6–7 strong ac. Wing hyaline, halter yellow. Legs with coxae and femora blackish brown, latter with pale knees; remainder of legs brown with apical part of tibiae I–III and tarsomeres I–III_1_ largely yellowish brown. Tibia II without ad and pd bristle pair. Surstylus with small split in dorsal and ventral lobe at apical 1/4; hypandrium straight (lateral view), ribbon-shaped, with acute apex (ventral view).

**Description**. **Male**. Body length: 2.0 mm (*n* = 1); wing length: 2.2 mm (*n* = 1), wing 0.3× as wide as long. **Head**. Face with epistoma metallic green in centre, clypeus mainly brilliantly bronze, both dusted greyish laterally; mainly parallel-sided, only slightly widening towards antennae and clypeus, as wide in middle as 2× postpedicel (length); epistoma about 2× as high as clypeus, latter as long as wide, bare. Frons metallic greenish blue, with strong whitish dusting. Occiput bronze, with slight greyish dusting, distinctly concave. Palpus small (not possible to measure reliably), ovoid, dark brown, apparently shining, with yellow pubescence and yellow ap bristle. Proboscis dark brown. Eye with red base colour and green reflection in more than lower 2/3, upper part violet red (with smaller ommatidia—hard to assess due to shrinking of eyes), bare. Postocular bristles white, lowermost quite long and gently curved, upper ones becoming shorter and erect (uppermost bristles missing). One pair of (missing) postocellar bristles. Antenna entirely black, with scape bare and pedicel with an apical crown of small bristles; postpedicel subcircular, about 0.8× as long as deep, and about 0.8× as long as scape and pedicel combined, with microscopic pubescence; stylus strictly apical, about 3.6× as long as first three antennal segments combined, with very short first segment, bare. **Thorax**. Mesonotum mainly metallic green on dorsum, and humeral and notopleural areas metallic violet blue, with strong whitish dusting; pleura metallic green, bronze to violet, with weak whitish dusting; scutellum greenish blue, with strong whitish dusting, with four bristles (no reliable measurements possible due to the lack of the median bristles). Thoracic bristles black. With four dc (one presutural, one sutural, two postsutural; most bristles lacking). A total of 6–7 ac, biseriate, reaching beyond second (sutural) dc, as long as distance between rows; three (one large black, two minute white) ant pprn, one external bas pprn, one strong sut ial, one npl, one spal, and one pal bristles. With about six minute pale setae between humeral and first dc bristle. Two white prothoracic bristles, with lower bristle considerably longer than upper one. **Wing**. Hyaline, with dark brown veins. Vein R_4+5_ smoothly sinuous, M_1_ gently curved, both distinctly converging towards wing apex; distance at wing apex less than 0.3× distance at crossvein dm-cu. Proximal section of M_1_ 0.9× as long as apical section. Proximal section of CuA_1_ 2.8× as long as apical section. CuA_x_ ratio: 0.7. Halter yellow, calypteral fringe white. **Legs**. Coxae I–III and femora I–III overall blackish brown, with pale knees, especially femora I–II; remainder of legs brown with apical part of tibiae I–III and tarsomeres I–III_1_ largely yellowish brown. With pale pubescence and bristles. Coxa I with whitish yellow pubescence, nearly exclusively on anterior face (lateral face nearly entirely bare), and four white ap bristles. Coxa II with white pubescence on anterior face, and one large white bristle on anterolateral margin. Coxa III with one white external bristle in centre. Trochanters brown, trochanter II with three minute white anterior bristles. Femur I blackish brown, with ap 1/5 yellow. Femur II dark brown, with more than ap 1/4 pale yellow. Femur III blackish brown, with less than ap 1/5 reddish yellow; femora largely devoid of bristles. Tibia I brown, paler on ap 1/4, without distinct bristles. Tibia II yellowish brown at basis to brownish yellow at apex, with four small ap bristles. Tibia III brown, paler at apex, with one yellow bristle at less than ap 1/4, slightly longer than tibia is deep, amid serration of short white setae on ap 1/2, and two indistinct av bristles with pd and vt serration of short white setae on ap 1/2. Tarsus I mainly brown, with taI_5_ dark brown; taI_1–4_ with apv crown of dark setae. Tarsus II with taII_1_ brownish yellow, darker from ap 1/3 onwards, with taII_2–5_ dark brown; taII_1–5_ with small dark vt setae and taII_1–4_ with apv crown of dark setae. Tarsus III with taIII_1_ yellowish brown, darker at apex, and taIII_2–5_ dark brown; taIII_2–4_ with apv crown of brown setae; taIII_1_ with rather blunt pv tooth at basis (MSSC). Ratio of femur/tibia/tarsomeres 1–5 in leg I: 8.4/8/4.4/2/1.4/1/1.1, in leg II: 8.3/8.6/5.3/2.4/1.5/0.9/1, and in leg III: 8.1/10.3/3/4/2.1/1/1. **Abdomen**. With seven pubescent segments. Tergites greenish bronze, with rather weak whitish pruinosity, with white pubescence and short bristles on posterior margins; bristles on sides of T_1_ erect and large. Sternites concolorous with T, with white, very short pubescence; S_2–5_ with blunt incision in middle of posterior margin, S_6_ entirely unsclerotised. Hypopygium incl. epandrium brownish black; hypandrium brownish yellow, rather wide, only slightly narrowing towards rather acute apex; with two small basal, and two large apicoventral epandrial setae, latter on short stalks; surstylus brown, gently curved ventralwards, split in ds and vt lobe at about apical 1/4, both with modified setae; cercus grey, rather small, elongate triangular, with two flattened ap bristles.

**Female**. Unknown.

**Type material. HOLOTYPE**: 1♂, **SPAIN**: Andalucía, Almería, Bayárcal (lush vegetation along mountain rivulet) (Mh_1), 37.0746, −3.0198, 1692 m, 2.viii.2015, SW, leg. Piluca Álvarez Fidalgo—sample cd: ES/2015/004a/PA [MNCN].

**PARATYPES**: **SPAIN**: 1♂, Castilla-La Mancha, Toledo, Argés, Guajaraz Reservoir (on rocks at reservoir) (Mh_2), 39.79655, −4.08379, 605 m, 26.iv.2017, SW, leg. Piluca Álvarez Fidalgo—sample cd: ES/2017/001/PA [MAPC].

**Distribution**. At present only recorded from central (Castilla-La Mancha) and southeastern Spain (Andalucía).

**Etymology**. The specific epithet “*hispanica*” refers to the country where the species has been discovered for the first time.

**Ecology**. Shared features of both capture sites suggest a preference of this species for open water (rivulet, reservoir) and possibly higher altitudes (605 m, 1692 m), but this remains to be confirmed by additional records.

**Figure 8:**
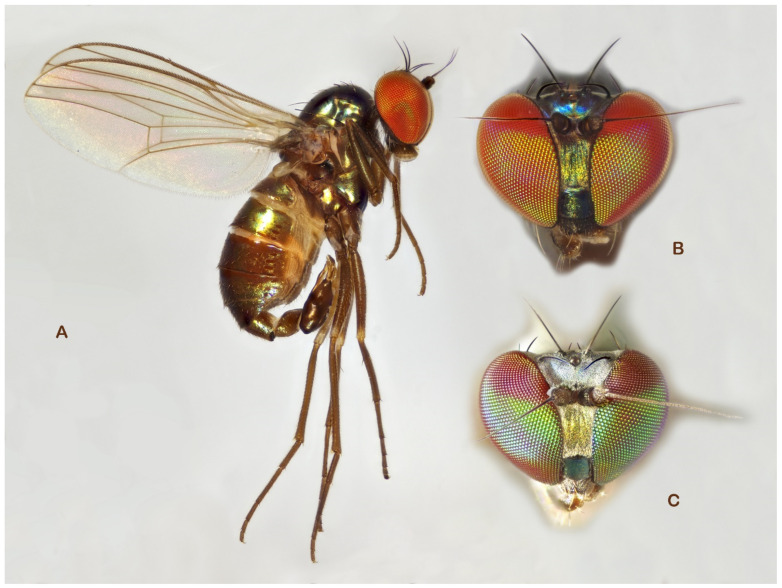
*Medetera lusitana* sp. nov. (**A**) male, general habitus; (**B**) male, head, in wet condition (frontal view); (**C**) male, head, dried superficially (frontal view) (photos: Andreas Stark).

**Figure 9:**
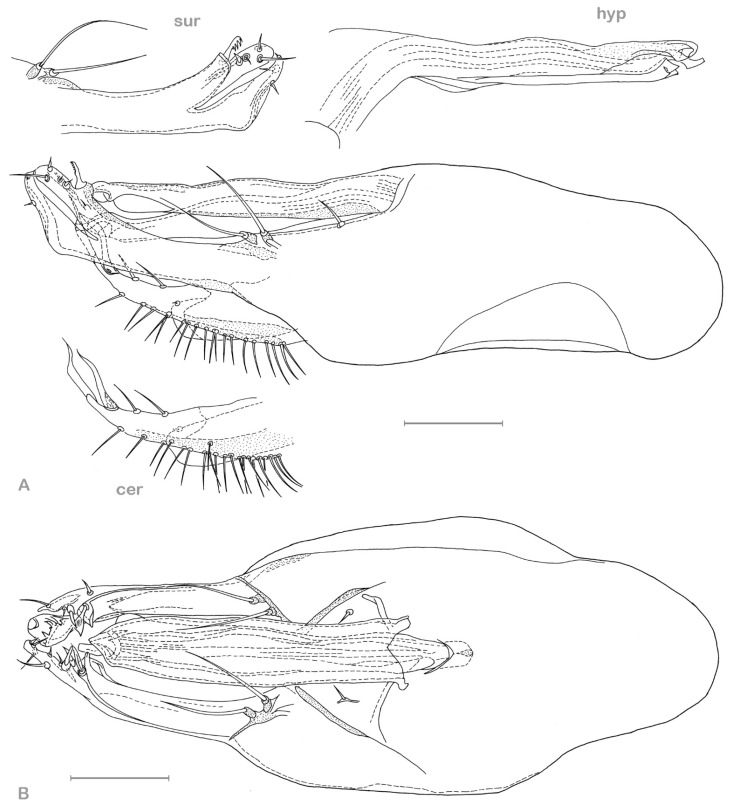
*Medetera lusitana* sp. nov. hypopygium (**A**) lateral view; (**B**) ventral view. Cer: cercus, hyp: hypandrium, sur: apical part of surstylus. Scale = 0.1 mm.

***Medetera lusitana*** Pollet sp. nov. (Figure 1D, Figure 2, Figure 8 and Figure 9)

urn:lsid:zoobank.org:act:0120BA70-872F-4B0F-B96A-FA6725595DD8

**Diagnosis**. Small but robust species, wing length 2.1–2.5 mm. Face with epistoma metallic green, strongly dusted yellowish, and clypeus brilliant green. Eye with red base colour and brilliant green reflection in lower 2/3, and dark violet in about upper 1/3 (with smaller ommatidia). Mesonotum (incl. scutellum) greenish blue with coppery area between dc rows extending upon prescutellar depression, with strong whitish dusting. Five dc, with first dc small, about 0.7× as long as second dc bristle; four strong ac; two spal bristles. Wing hyaline; halter dark. Legs overall blackish brown, and femora I–III with narrow reddish yellow knees. Tibia II with ad and pd bristle pair. Hypandrium slightly undulating ventrally (lateral view), stout, widest in middle with acute apex, without lateral hooks at basal 1/3 (ventral view).

**Description**. **Male**. Body length: 2.2–2.5 mm; wing length: 2.1–2.5 mm, wing 0.4× as wide as long. **Head**. Face with epistoma metallic green, strongly dusted yellowish and clypeus brilliant green; narrowing below antennae, parallel-sided in middle (1/3 of eye width) and slightly diverging at clypeus; epistoma with 2–3 shallow diverging furrows, clypeus about as long as wide, bare. Frons metallic violet blue, with strong whitish dusting. Occiput violet blue, strongly dusted, distinctly concave. Palpus small, 1/6 of eye height, ovoid, dark brown, shining, with yellow pubescence and brown ap bristle. Proboscis dark brown. Eye with red base colour and brilliant green reflection in lower 2/3, and dark violet in about upper 1/3 (with smaller ommatidia), bare. Most postocular bristles white, rather long, gently curved, with uppermost three distinctly shorter and darker. One pair of black postocellar bristles. Antenna entirely black, with scape bare and pedicel with apical crown of small bristles; postpedicel subcircular, with small blunt apv process, about as long as deep, and about 0.6× as long as scape and pedicel combined, with microscopic pubescence; stylus apical, above small blunt process, about 4× as long as first three antennal segments combined, with very short first segment, bare. **Thorax**. Mesonotum (incl. scutellum) greenish blue with coppery area between dc rows extending upon prescutellar depression, with strong whitish dusting; pleura metallic green, with weaker whitish dusting. Thoracic bristles black. Five strong dc (two presutural, one sutural, two postsutural), first dc 0.7× as long as second dc, posterior ones increasing in length towards scutellum. Four ac, biseriate, reaching until third dc, rather strong, about 1.5–2× as long as distance between rows; three (one large black, two minute white) ant pprn, one external bas pprn, one (minute, ac-sized) psut ial, one rather strong sut ial, one npl, two spal, and one pal bristles. Scutellum with four bristles, lateral ones strong, about 0.7× as long as median ones. With 2–3 white prothoracic bristles, lower bristle about 2× as long as upper bristles. In some specimens, about eight minute setae between ant pprn bristle and first dc. **Wing**. Hyaline, with dark brown veins. Vein R_4+5_ smoothly sinuous, M_1_ gently curved, both distinctly converging towards wing apex, and sometimes slightly diverging at wing apex; distance at wing apex 0.3× distance at crossvein dm-cu. Proximal section of M_1_ 0.9× as long as apical section. Proximal section of CuA_1_ 3.0× as long as apical section. CuA_x_ ratio: 0.7. Halter black, both shaft and knob, calypteral fringe brown. **Legs**. Overall blackish brown, femora I–III with narrow reddish yellow knees, especially femur II. With pale vt and dark ds bristles. Coxae I–II blackish brown, coxa I with whitish yellow pubescence nearly exclusively on anterior face (lateral face nearly entirely bare), and four yellow ap bristles. Coxa II with whitish yellow pubescence only on anterior face, and three whitish yellow bristles on anterolateral margin, with the apicalmost bristle strongest. Coxa III blackish brown, with one whitish yellow external bristle, inserted at basal 2/5 in middle. Trochanters blackish brown, trochanter II with two minute brown anterior bristles. Femur I blackish brown, with paler extreme apex. Femora II–III blackish brown, with reddish yellow apex; femur II with very short pale av and vt setae. Femur III with some short yellow vt setae on apical 1/4, and with basodorsal row of strong brown bristles on mainly basal 2/5, with four erect basal bristles, longest ones as long as femur is deep. Tibiae I–III blackish brown. Tibia I without distinct bristles. Tibia II blackish brown, with one ad and one pd bristle at less than basal 1/3 (pd bristle slightly more apical than ad bristle), former 1.5× as long as tibia is deep and latter slightly longer than tibia is deep; with five ap bristles, two vt ones strongest. Tibia III with apicodorsal notch and weak ds serration, including one strongly inclined yellow bristle at ap 1/5, about as long as tibia is deep, and two indistinct av bristles; with pd serration of short white setae, especially on apical 1/2. Tarsi I–III blackish brown. Tarsus I: taI_1_ with minute yellow vt setae, and taI_1–4_ with apv crown of dark setae. Tarsus II: taII_1–5_ with several small dark vt setae, and taII_1–4_ with apv crown of dark setae. Tarsus III with short yellow setae mainly on taIII_1–2_, 2 longer vt brown setae at apex of taIII_1_, and taIII_1–4_ with apv crown of dark setae. Ratio of femur/tibia/tarsomeres 1–5 in leg I: 10.1/9.3/4.7/1.9/1.5/1/1.3, in leg II: 10.3/10.3/5.7/2.5/1.8/1/1, and in leg III: 8.8/11.6/3.3/4/2.1/1/1. **Abdomen**. With seven pubescent segments. Tergites metallic green, with weak whitish pruinosity, with dark (brown) pubescence and short bristles on posterior margins; bristles on sides of T_1_ pale brown to yellow, erect and large. Sternites dark brown, with weak metallic green reflection, with yellow, very short pubescence; S_2–5_ with blunt incision in middle of posterior margin, S_6_ entirely unsclerotised. Hypopygium incl. epandrium brownish black; hypandrium reddish brown, wide, widest in middle, with triangular apex; with one small basal, and two larger apicoventral epandrial setae, latter on short stalks; surstylus brownish yellow, rather robust, with clear ds and vt lobe from about apical 1/3; ventral surstylar lobe with modified seta; cercus pale brown, rather small, elongate triangular, with two ap flattened bristles.

**Female**. As male, except for body length: 2.3–2.8 mm; wing length: 2.3–2.7 mm. Face 1/3 of eye width in middle. Wing with proximal section of CuA_1_ 2.6× as long as apical section. Abdomen with five pubescent segments; segments 6–11 telescopic, and 12th segment with two black needle-like acanthophorites and two equal-sized brown cerci. Abdomen with metallic tergites, from greenish bronze to bluish green, with weak whitish pruinosity; sternites coloured as in male but S_2–5_ complete. Ratio of femur/tibia/tarsomeres 1–5 in leg I: 9/8.3/4.1/1.8/1.3/1/1.2, in leg II: 8.6/8.7/4.4/2.2/1.4/0.8/1, and in leg III: 8.2/10.7/2.8/3.7/1.9/1/1.

**Type material. HOLOTYPE**: 1♂, **PORTUGAL**: Alto Alentejo, Santa Maria de Marvão (Mediterranean shrubland with big rocks scattered across the landscape and forest patches of *Quercus pyrenaica* and *Quercus suber*) (Ml_2), 39°23′49.2″ N, 7°21′51.4″ W, 610 m, 21.ix.2014, SW, leg. Ana Rita Gonçalves—sample cd: PT/2014/176/AG [RBINS, IG: 34520/005].

**PARATYPES: PORTUGAL**: 7♂, same data as holotype; 1♂, 1♀, Beira Alta, Lapa dos Dinheiros (small stream in very rocky mountainous landscape with many shrubs) (Ml_3), 40°21′50.1″ N, 7°39′07.5″ W, 1547 m, 29.viii.2015, SW, leg. Rui Andrade—sample cd: PT/2015/330/RA; 1♂, Lapa dos Dinheiros, Porto do Boi (rocky (granitic) mountain stream, surrounded by low shrublands) (Ml_4), 40°22′17.69″ N, 7°40′33.16″ W, 1004 m, 4.ix.2014, SW, leg. Ana Rita Gonçalves—sample cd: PT/2014/200/AG; 4♂, 1♀, Loriga, Praia Fluvial de Loriga (rocky mountain river) (Ml_5), 40°19′41.26″ N, 7°40′36.71″ W, 890 m, 3.ix.2014, SW, leg. Ana Rita Gonçalves—sample cd: PT/2014/204/AG; 1♀, São Pedro (Manteigas) (riparian corridor in mountainous site) (Ml_6), 40°22′10.4″ N, 7°33′04.4″ W, 986 m, 22.ix.2013, SW, leg. Ana Rita Gonçalves—sample cd: PT/2013/272/AG; 1♂, 1♀, same data, SW, leg. Rui Andrade—sample cd: PT/2013/186/RA; 1♀, São Pedro (Manteigas), Covão d’Ametade (depression of glacial origin with small stream at bottom) (Ml_7), 40°19′42.2″ N, 7°35′17.4″ W, 1450 m, 22.ix.2013, SW, leg. Ana Rita Gonçalves—sample cd: PT/2013/282/AG; 1♀, São Pedro (Manteigas), Nave de Santo António (mountain plateau with herbaceous plants and shrubs) (Ml_8), 40°19′07.0″ N, 7°34′37.4″ W, 1539 m, 21.ix.2013, SW, leg. Ana Rita Gonçalves—sample cd: PT/2013/165/AG; 1♂, 2♀, Trinta (riparian corridor in a mountainous site crossing oak forest) (Ml_9), 40°30′31.1″ N, 7°22′21.7″ W, 751 m, 22.ix.2013, SW, leg. Ana Rita Gonçalves—sample cd: PT/2013/208/AG; 1♂, Beira Baixa, Cortes do Meio, Alto da Pedrice (humid shrubland) (Ml_10), 40°18′38.83″ N, 7°34′35.14″ W, 1645 m, 2.ix.2014, HC, leg. Ana Rita Gonçalves—sample cd: PT/2014/376/AG; 2♂, 4♀, Douro Litoral, Avintes, Parque Biológico de Gaia (PBG) (park of ca. 35 ha with small Febros river and many trees within a strongly altered landscape) (Ml_11), 41°05′57.9″ N, 8°33′35.0″ W, 60 m, 15.v.2009, HC, leg. Rui Andrade—sample cd: PT/2009/128/RA; 1♂, same site, 1.vi.2013, SW, leg. Rui Andrade—sample cd: PT/2013/100/RA; 2♂, 1♀, Minho, Castro Laboreiro, Cainheiras (mountainous mosaic landscape of grasslands and forests crossed by a small river) (Ml_12), 42°01′32.8″ N, 8°08′18.8″ W, 980 m, 10.ix.2016, SW, leg. Rui Andrade—sample cd: PT/2016/392/RA; 1♂, Trás-os-Montes e Alto Douro, Algoso, Vale de Algoso (village with small plots of agricultural land and surrounded by a Mediterranean shrubland) (Ml_13), 41°29′52.4″ N, 6°32′19.9″ W, 575 m, 5.x.2015, SW, leg. Rui Andrade and Ana Gonçalves—sample cd: PT/2015/379/RA&AG; 1♀, Bilhó (waterfall on mountain river in pine forest) (Ml_14), 41°23′48.7″ N, 7°50′53.3″ W, 695 m, 26.vi.2013, SW, leg. Rui Andrade—sample cd: PT/2013/036/RA; 1♂, 2♀, Carrazedo (meadows in oak forest) (Ml_15), 41°45′51.7″ N, 6°54′06.2″ W, 916 m, 19.vi.2015, SW, leg. Rui Andrade—sample cd: PT/2015/313/RA; 2♂, Ermelo, Varzigueto (wooded banks of mountain river crossing meadows) (Ml_16), 41°22′44.0″ N, 7°51′14.4″ W, 750 m, 25.vi.2013, SW, leg. Rui Andrade—sample cd: PT/2013/008/RA; 2♂, França, Aldeia de Montesinho (permanent pastures (Lameiros) in a mountainous area) (Ml_17), 41°56′33.0″ N, 6°45′51.0″ W, 1049 m, 7.viii.2014, SW, leg. Rui Andrade—sample cd: PT/2014/249/RA; 1♂, Pitões das Júnias (oak forest and Mediterranean shrubland) (Ml_18), 41°49′48.7″ N, 7°57′01.3″ W, 965 m, 10.ix.2012, SW, leg. Rui Andrade—sample cd: PT/2012/051/RA. **SPAIN**: 1♂, Galicia, Ourense, Baltar, San Martiño (farmland near river) (Ml_1), 41°54′2.5″ N, 7°46′49.4″ W, 1030 m, 13.ix.2008, SW, leg. J.L. Camaño Portela—sample cd: ABIGA/16038 [all RBINS, IG: 34520/006; and MAPC].

**Distribution**. At present only known from northern and northeastern Portugal (Minho, Trás-os-Montes e Alto Douro, Douro Litoral, Beira Alta, Beira Baixa, Alto Alentejo) and northwestern Spain (Ourense).

**Etymology**. The specific epithet “*lusitana*” is derived from Lusitania, the name of a Roman province in the Iberian Peninsula, which encompasses most of modern Portugal. It refers to the country (Portugal) where the species seems to reach its main distribution.

**Ecology**. With Parque Biológico de Gaia (strongly impacted lowland area in Avintes at 60 m a.s.l.) as only exception, *M. lusitana* sp. nov. has only been collected at higher altitudes, between 575 m and 1645 m a.s.l., at rather humid spots (near waterfalls, small streams, riparian corridors) in an otherwise mainly open dry landscape with shrubs and scattered trees. Rocks were often present in these sites (see also *M. aglaops* sp.n.).

**Figure 10:**
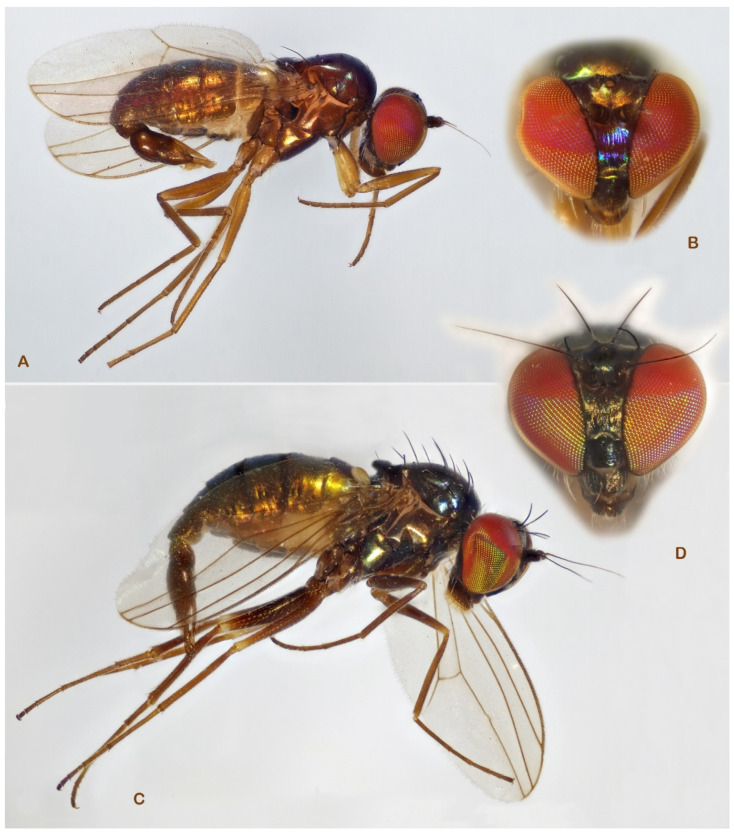
*Medetera* species (**A**,**B**) *Medetera parva* sp. nov., male (**A**) general habitus; (**B**) head (frontal view); (**C**,**D**) *Medetera rectipyga* sp. nov., male (**C**) general habitus; (**D**) head (frontal view) (photos: Andreas Stark).

**Figure 11:**
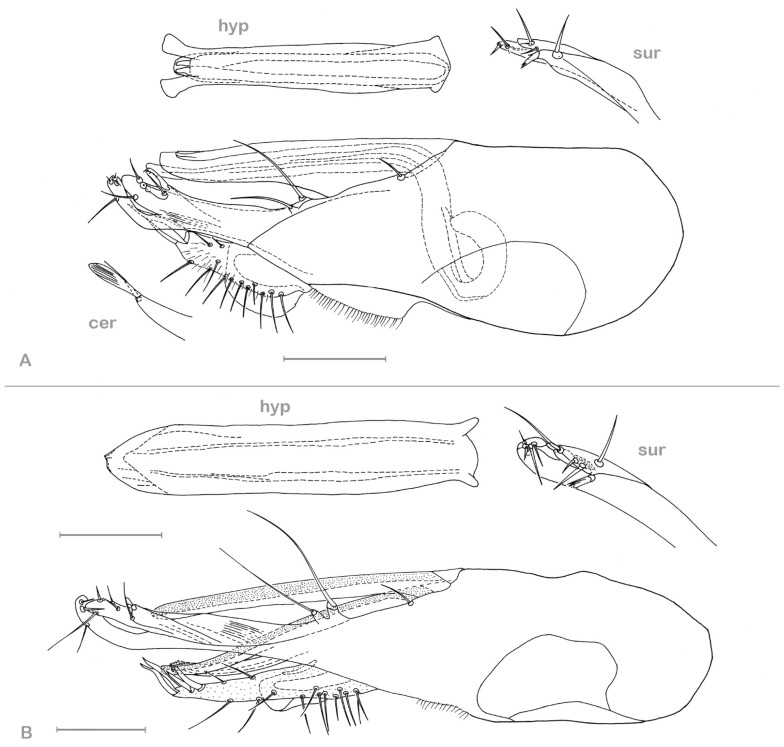
*Medetera* hypopygia, lateral view (**A**) *Medetera parva* sp. nov.; (**B**) *Medetera rectipyga* sp. nov. Cer: cercus, hyp: hypandrium, sur: apical part of surstylus. Scale = 0.1 mm.

***Medetera parva*** Pollet sp. nov. (Figure 2, Figure 10A,B and Figure 11A)

urn:lsid:zoobank.org:act:AEAF6AD1-2909-4989-B270-B6228EE785B5

**Diagnosis**. Very small species, wing length 1.7–1.8 mm. Face with epistoma brilliant violet, and clypeus brilliant bluish green, both separated by narrow grey pollinose zone. Eye with red base colour, with three zones, middle zone purely red, and upper (with smaller ommatidia) and lower zones with greenish reflection. Mesonotum mainly bluish green on dorsum, with distinct blue violet humeral and notopleural zones, with strong whitish dusting. Three–four dc; ac absent. Wing very slightly infuscate; halter yellow. Legs overall mainly yellowish to pale brown, coxae II–III entirely and coxa I in part brown at basis, and tarsi I–III only slightly darker towards apex. Tibia II without ad and pd bristle pair. Surstylus straight and short, with split at apical 1/3; hypandrium straight dorsally (lateral view), with two lateral apical processes (ventral view).

**Description**. **Male**. Body length: 1.8–2.0 mm (*n* = 3); wing length: 1.7–1.8 mm (*n* = 3), wing 0.4× as wide as long. Head. Face with epistoma brilliant violet, and clypeus brilliant bluish green, both separated by narrow grey pollinose zone; mainly parallel-sided, only slightly widening towards antennae and clypeus, 1/2 of eye width in middle, epistoma 1.5× as high as clypeus, latter about as long as wide, bare. Frons metallic blue, with strong whitish dusting. Occiput bronze, with slight greyish dusting, distinctly concave. Palpus small, 1/5 of eye height, ovoid, dark brown, with grey dusting, with yellow pubescence and yellow ap bristle. Proboscis dark brown. Eye with red base colour, with three zones, middle zone purely red, and upper (with smaller ommatidia) and lower zones with greenish reflection, bare. Postocular bristles all white, with lowermost quite long and gently curved, and uppermost short and erect. One pair of minute brown postocellar bristles. Antenna entirely dark brown, with scape bare and pedicel with an apical crown of small bristles; postpedicel subquadrate (with ventral apex more protruding), about as long as deep, distinctly smaller than pedicel, and 0.7× as long as scape and pedicel combined, with microscopic pubescence; stylus apical, about 3.4× as long as first three antennal segments combined, with very short first segment, bare. **Thorax**. Mesonotum mainly bluish green on dorsum, with distinct blue violet humeral and notopleural zones, with strong whitish dusting; pleura metallic green, bronze to violet, with weak whitish dusting; scutellum greenish blue, with strong whitish dusting, with four bristles (but lacking). Thoracic bristles dark brown. With 3–4 dc (one small sutural, two larger postsutural, and sometimes one small presutural). Ac absent; seemingly two (one large dark brown, one minute white) ant pprn, one external bas pprn, one npl, one spal, and one pal bristles. With 2–3 white prothoracic bristles, with lower bristle longest. **Wing**. Very slightly infuscate. Vein R_4+5_ and M_1_ gently curved (latter sometimes nearly straight), distinctly converging towards wing apex; distance at wing apex 0.3× that at crossvein dm-cu. Proximal section of M_1_ 0.8× as long as apical section. Proximal section of CuA_1_ 2.5× as long as apical section. CuA_x_ ratio: 0.7. Halter yellow, calypteral fringe white. **Legs**. Overall, mainly yellowish to pale brown, coxae II–III entirely and coxa I in part brown at basis, and tarsi I–III only slightly darker towards apex. With pale pubescence and bristles. Coxa I mainly yellowish brown, brown on outer 1/2, with white, rather strong and erect pubescence on entire face, and four white ap bristles. Coxae II–III blackish brown; coxa II with white pubescence on anterior face, and three white bristles on anterolateral margin, with the apicalmost bristle strongest; coxa III with one white external bristle at ap 1/2 in anterior 1/3. Trochanters brown, trochanter II with three minute white anterior bristles. Femur I yellowish to pale brown, slightly infuscate dorsally, paler at apex, with one pv row of minute erect white setae along entire length. Femora II–III yellowish to pale brown, with narrow paler knee; femur III with one av row of minute inclined white setae along entire length. Tibiae I–III yellowish to pale brown. Tibia I without distinct bristles. Tibia II with four small ap bristles. Tibia III with one yellow bristle at about apical 1/7, with weak serration of short white setae along entire length, and two indistinct av bristles. Tarsi I–III yellowish brown to brown, often darker towards apex. Tarsus II: taII_1–4_ with apv crown of brown setae. Tarsus III: taIII_1_ with indistinct blunt pv tooth at basis (MSSC). Ratio of femur/tibia/tarsomeres 1–5 in leg I: 9.2/8.6/4.4/2.2/1.5/1/1.2, in leg II: 9.3/9.5/5.6/2.7/2.1/1/1, and in leg III: 7.5/9.5/2.6/3.8/2/1/1. **Abdomen**. With 7 pubescent segments. Tergites greenish bronze, with rather weak whitish pruinosity, with white pubescence and short bristles on posterior margins; bristles on sides of T1 more erect and large. Sternites concolorous with T, with white, very short pubescence; S_2–5_ with blunt incision in middle of posterior margin, S_6_ entirely unsclerotised. Hypopygium with epandrium brownish black, rather robust; hypandrium reddish yellow, slightly narrowing towards apex, with two lateral square ap processes; with one small basal, and two larger apicoventral epandrial setae, latter on very short stalks; surstylus reddish yellow to yellowish brown with darker basis, robust and rather short, separated from epandrium by suture, split in ds and vt lobe at ap 1/3; ventral lobe with one modified seta; cercus rather small, elongate triangular, with two ap flattened bristles.

**Female**. Unknown.

**Type material. HOLOTYPE**: 1♂, **ITALY**: Sardinia, Carbonia-Iglesias, Iglesias, Marganai (Plot Conecofor SAR1) (Mp_1), 39.354434° N, 8.574515° E, 700 m, 16.vi–14.vii.2005, MT, leg. G. Chessa—sample cd: IT/2006/031/GN (Conecofor Programme—CNBF 2003) [MZUR].

**PARATYPES**: **ITALY**: 1♂, same data as holotype; 1♂, same site, 14.vii–5.viii.2005, MT, leg. G. Chessa—sample cd: IT/2006/008/GN (Conecofor Programme—CNBF 2003) [both MAPC].

**Distribution**. At present only known from Sardinia (Carbonia-Iglesias) (Italy).

**Etymology**. The specific epithet “*parva*” is derived from latin “*parvus*” and refers to the small size of this *Medetera* species.

**Ecology**. The type locality is the permanent level II monitoring plot of the CONECOFOR-ICP forest network [22], adjacent to an ancient holm oak coppice on a south-facing slope, belonging to the *Viburnum-Quercetum ilicis* association. The shrub layer is characterised by *Ilex aquifolium* [21].

***Medetera rectipyga*** Pollet sp. nov. (Figure 2, Figure 10C,D and Figure 11B)

urn:lsid:zoobank.org:act:A558401F-BF87-413D-AE84-175E235CDF36

**Diagnosis**. Small but robust species, wing length 2.0–2.4 mm. Face with epistoma metallic bronze in centre, clypeus mainly brilliantly bronze or green, both dusted greyish laterally. Eye with red base colour and green reflection in lower 2/3–1/2, purple red in upper 1/3–1/2 (with smaller ommatidia). Mesonotum (incl. scutellum) reddish bronze in middle of dorsum, with distinctly metallic violet blue humeral and notopleural areas, with strong greyish dusting. Three dc; ac absent. Legs overall mainly pale brown to brown, with coxae I–III, femora I and III blackish brown, and femora I–III with paler knees. Tibia II without ad and pd bristle pair. Surstylus very slender and straight, with split at about ap 1/3; hypandrium rather straight dorsally (lateral view), wide, parallel-sided with rounded apex, without raised basis in lateral view (ventral view).

**Description**. **Male**. Body length: 2.0–2.5 mm; wing length: 2.0–2.4 mm, wing 0.4× as wide as long. **Head**. Face with epistoma metallic bronze in centre, clypeus mainly brilliant bronze or green, both dusted greyish laterally; narrowing below antennae, parallel-sided in middle (1/2 of eye width) and slightly diverging at clypeus; epistoma with one small central bulge, 1.5× as high as clypeus, latter as long as wide, both bare. Frons metallic greenish blue, with weak greyish dusting. Occiput bronze, with slight greyish dusting, distinctly concave. Palpus small, 1/7 of eye height, ovoid, dark brown, shining, with yellow pubescence and yellow ap bristle. Proboscis dark brown. Eye with red base colour and green reflection in lower 2/3–1/2, purple red in upper 1/3–1/2 (with smaller ommatidia), bare. Postocular bristles all white, with lowermost quite long and gently curved, and uppermost short and erect. One pair of minute brown postocellar bristles. Antenna entirely black, with scape bare and pedicel with apical crown of small bristles; postpedicel subquadrate (with ventral apex slightly protruding), slightly longer than deep, and about 0.8× as long as scape and pedicel combined, with microscopic pubescence; stylus apical (dorsad of ventral process), about 4× as long as first three antennal segments combined, with very short first segment, bare. **Thorax**. Mesonotum (incl. scutellum) reddish bronze in middle of dorsum, with distinctly metallic violet blue humeral and notopleural areas, with strong greyish dusting; pleura metallic greenish bronze, with weak greyish dusting. Thoracic bristles black. With three strong dc (one presutural, two postsutural), increasing in length towards scutellum. Ac absent. Two (one large black, one minute white) ant pprn, one external bas pprn, one rather strong sut ial, one npl, one spal, and one pal bristles. Scutellum with four bristles, lateral ones rather small, about 0.4× as long as median ones. With 2–3 white prothoracic bristles, with lower bristle longest. **Wing**. Slightly infuscate, with brown veins. Vein R_4+5_ smoothly sinuous, M_1_ gently curved, both distinctly converging towards wing apex, parallel at wing apex; distance at wing apex 0.4× that at crossvein dm-cu. Proximal section of M_1_ 0.9× as long as apical section. Proximal section of CuA_1_ 2.9× as long as apical section. CuA_x_ ratio: 0.7. Halter yellowish, at most slightly infuscate, calypteral fringe yellow. **Legs**. Overall mainly pale brown to brown, with coxae I–III, femora I and III blackish brown, and femora I–III with paler knees, most distinct in femur II. Pubescence and bristles pale. Coxae I–III blackish brown, with coxa I paler on apical 1/2, latter with white pubescence, nearly exclusively on anterior face, and four white ap bristles. Coxa II with white pubescence on anterior face, and one large white bristle on anterolateral margin. Coxa III with one white external bristle in centre. Trochanters brown, trochanter II with three minute white anterior bristles. Femur I blackish brown, with extreme apex paler. Femur II dark brown, with about ap 1/4 paler. Femur III blackish brown, with extreme apex reddish yellow; femora largely devoid of bristles. Tibiae I–III dark brown. Tibia I without distinct bristles. Tibia II with four small ap bristles. Tibia III with one yellow bristle at ap 1/5, slightly longer than tibia is deep, and two indistinct av bristles. Tarsi I–III dark brown. Tarsus I with taI_1–4_ with apv crown of dark setae. Tarsus II with taII_1–5_ with several small dark vt setae, and taII_1–4_ with apv crown of dark setae. Tarsus III with taIII_2–5_ with several small dark vt setae, and taIII_2–4_ with apv crown of dark setae; taIII_1_ with rather acute pv tooth at basis (MSSC). Ratio of femur/tibia/tarsomeres 1–5 in leg I: 7.9/7.7/4.1/2.1/4.1/1/1.1, in leg II: 8.9/9.4/5.7/2.6/1.8/1/1, and in leg III: 6.9/8.9/2.5/3.8/2/1/1. **Abdomen**. With seven pubescent segments. Tergites greenish bronze, with rather strong yellowish grey pruinosity, with white pubescence and short bristles on posterior margins; bristles on sides of T_1_ erect and large. Sternites concolorous with T, with white, very short pubescence; S_2–5_ with blunt incision in middle of posterior margin, S_6_ entirely unsclerotised. Hypopygium incl. epandrium brownish black; hypandrium reddish brown, rather wide, parallel-sided with rounded apex; with one small basal, and two larger apicoventral epandrial setae, latter on short stalks; surstylus dark brown, conspicuously straight, rather slender, with split in ds and vt lobe at about ap 1/3, with most setae unmodified; cercus grey, rather small, elongate triangular, with two flattened ap bristles.

**Female**. As male, except for body length: 2.0–2.3 mm (*n* = 4); wing length: 2.0–2.2 mm (*n* = 4), wing 0.3× as wide as long. Proximal section of M_1_ 0.8× as long as apical section. Proximal section of CuA_1_ 2.7× as long as apical section. Abdomen with five pubescent segments; segments 6–11 telescopic, and 12th segment with two black needle-like acanthophorites and two equal-sized brown cerci. Sternites coloured as in male but S_2–5_ complete. Ratio of femur/tibia/tarsomeres 1–5 in leg I: 8/7.7/4/2/1.4/1/1.1, in leg II: 8.3/8.8/5.2/2.5/1.6/1/1, and in leg III: 7.6/9.8/2.5/3.9/2/1/1.

**Type material. HOLOTYPE**: 1♂, **SPAIN**: Cataluña, Lleida, Pallars Sobirà, Soriguera (mountain slope covered mostly by shrubs, grasses and several flowering Apiaceae) (Mr_1), 42°22′22.6″ N, 1°12′07.5″ E, 1560 m, 4.viii.2014, SW, leg. Rui Andrade—sample cd: ES/2014/250/RA [RBINS, IG: 34520/007].

**PARATYPES**: **SPAIN**: 1♂, 1♀, same data as holotype; 3♀, Pallars Sobirà, Sort (Llessui) (mountainous location with a mosaic of pastures and forests) (Mr_2), 42°27′03.6″ N, 1°04′14.6″ E, 1410 m, 3.viii.2014, SW, leg. Rui Andrade and Ana Gonçalves—sample cd: ES/2014/167/RA&AG; 4♂, same site, 4.viii.2014, SW, leg. Rui Andrade and Ana Gonçalves—sample cd: ES/2014/216/RA&AG. **FRANCE**: 1♂, Provence-Alpes-Côte d’Azur, Alpes-Maritimes, Gourdon, Riou de Gourdon (on flagstones along a stream) (Mr_3), 43.72186, 6.96490, 750 m, 4.vi.2021, HC, leg. Frédéric Belin—sample cd: FR/2021/050/FB; 2♂, Saint-Vallier-de-Thiey (on flagstones in garrigue) (Mr_4), 43.69925, 6.80283, 700 m, 9.vi.2022, HC, leg. Frédéric Belin—sample cd: FR/2022/069/FB; 1♂, Vence, chemin de la Gaude (on a large rock in garrigue) (Mr_5), 43.71860, 7.14326, 140 m, 25.v.2021, HC, leg. Frédéric Belin—sample cd: FR/2021/043/FB [all RBINS, IG: 34520/008; and MAPC].

**Distribution**. At present only known from northeastern Spain (Cataluña) and southeastern France (Dept Alpes-Maritimes, Provence-Alpes-Côte d’Azur).

**Etymology**. The specific epithet “*rectipyga*” refers to the straight slender shape of the surstylus, which is an important diagnostic feature in this species.

**Ecology**. In northeastern Spain, *M. rectipyga* sp. nov. has been collected at higher altitudes (1410–1560 m) in open mosaic habitats, mostly with grasses and shrubs. In southeastern France, it has indifferently been collected on rocks in different biotopes, from garrigue (dry open areas with shrubs) to stream banks.

### 3.2. Key to Males of New Mediterranean Medetera Species

For the identification of known *Medetera*, see [2] in combination with [31,32,33].

1 Tibia II with one pair of ad and pd bristles ........................................................................................................................................................................................2

- Tibia II without ad-pd bristle pair.........................................................................................................................................................................................................4

2 Eyes uniformly red, without dorsal violet part. Mesonotum uniformly metallic green–bronze (Figure 6A). Epistoma green, heavily dusted whitish. Surstylus with indistinct apical split. Hypopygium, see Figure 7A..............................................................................................................................................................................................***Medetera corsicana*** sp. nov. (Corsica)

- Eyes with red base colour and brilliant green reflection in lower 2/3, and dark violet colour in about upper 1/3.....................................................................3

3 Five dc, with first dc small, about 0.7× as long as second dc bristle. Four ac, strong, about 1.5–2× as long as distance between rows. Two spal bristles. Knob of halter dark (Figure 1D). Wing hyaline. Clypeus brilliantly green (Figure 8B). Hypandrium stout, without lateral hooks at basal 1/3. Hypopygium, see Figure 9A,B..................................................................................................................................***Medetera lusitana*** sp. nov. (Portugal, Spain)

- Five dc, with first dc very small, less than 0.5× as long as second dc bristle. More than 6 ac, microscopic. One spal bristle. Halter yellow (Figure 1A,C). Wing slightly smoked brownish. Clypeus strongly dusted greyish (Figure 1B and Figure 3B). Hypandrium slender, with lateral hooks at basal 1/3. Hypopygium, see Figure 4A,B and Figure 5..........................................................................................................................................................................................................................................................................................................................................................................................***Medetera aglaops*** sp. nov. (Portugal, Spain, France (mainland), Corsica, Sardinia)

4 Very small species, wing length less than 2.0 mm. Legs incl. coxa I and femora mainly yellowish brown (Figure 10A). Hypandrium with two lateral apical processes. Surstylus short and robust. Hypopygium, see Figure 11A................................................................................................................................................................................................***Medetera parva*** sp. nov. (Sardinia)

- Small species, wing length at least 2.0 mm. All coxae and femora blackish brown (Figure 6B,C and Figure 10D). Hypandrium without lateral apical processes. Surstylus more slender.........................................................................................................................................................................................................5

5 Six strong ac. Mesonotum mainly metallic green on dorsum with humeral and notopleural areas metallic violet blue (Figure 6C). Eyes with red base colour and green reflection in more than lower 2/3, upper part violet red. Epistoma metallic green in centre, with sides dusted. Hypopygium, see Figure 7C....................................................................................................................................................................................***Medetera hispanica*** sp. nov. (Spain)

- At most three microscopic ac, or ac absent. With other combination of characters........................................................................................................................6

6 Mesonotum uniformly bronze (Figure 6B). Eye with red base colour and green reflection in lower 4/5 and reddish purple in upper 1/5. A few ac mostly present. Hypandrium with raised basis (lateral view), with tulip-shaped apex (ventral view). Surstylus rather short, gently curved ventrally. Hypopygium, see Figure 7B.................................................................................................................................................***Medetera gibbosipyga*** sp. nov. (Spain)

- Mesonotum reddish bronze in middle of dorsum, with distinctly metallic violet blue humeral and notopleural areas (Figure 10D). Eye with red base colour and brilliant green reflection in lower 2/3–1/2, and violet in about upper 1/3–1/2. Ac absent. Hypandrium without raised basis (lateral view), wide and parallel-sided (ventral view). Surstylus very slender and straight. Hypopygium, see Figure 11B...................................................................................................................................................................................***Medetera rectipyga*** sp. nov. (Spain, France)

## 4. Discussion

Establishing the identity of many *Medetera* species is very often hampered by a lack of decisive diagnostic features. Indeed, most *Medetera* males do not feature specific Male Secondary Sexual Characters, which are common in other dolichopodid lineages and a valuable tool for identification. With the exception of only a few species, in the Palaearctic, females of most *Medetera* species cannot be identified with absolute certainty. Negrobov and Stackelberg [31,32] and Negrobov [33] provided a key to both sexes but many couplets end with male genital characters which excludes the identification of females. Even the most recent comprehensive key by Negrobov and Naglis [2] only included the male sex. As demonstrated here, part of the European *Medetera* diversity still remains to be explored, especially in the south. Moreover, this certainly holds true for most of the eastern parts of the Palaearctic as well. As females are often collected in larger numbers than males, there is a fair chance that some of these new species are only represented by female specimens in samples. For that reason, we considered a key to females (not only for the seven species treated here but for any *Medetera* fauna) unreliable, until more useful and reliable diagnostic characters are available. Barcoding might represent a promising tool for this purpose. In fact, once female specimens have been identified in this way, the search for reliable characters (also biometrics) can be seriously pursued.

Despite this poor set of diagnostic characters, we are confident that the seven species described here are new to science. In fact, none of them matches a species in the key by Negrobov and Naglis [2]. All four of our species without the ad–pd bristle pair on the mid tibia show pale postoculars, such as *M. glaucella* Kowarz, 1878 and *M. glaucelloides* Naglis, 2013, but clearly differ from either of these species. In addition, they also differ from *M. storai* Frey, 1935 (endemic to the Canary Islands) by a strongly pollinose mesonotum, size (wing length >1 mm) and CuA_x_ ratio (<1.0). In addition, *M. storai* does not seem to have a particular eye colour pattern. Both sexes of *M. aglaops* sp. nov., *M. gibbosipyga* sp. nov., *M. lusitana* sp. nov. and *M. rectipyga* sp. nov. show a distinct eye colour pattern. This character can thus not be regarded as a Male Secondary Sexual Character and most presumably does not play a role in courtship behaviour. Thus far, no females of *M. corsicana* sp. nov., *M. hispanica* sp. nov., or *M. parva* sp. nov. have been detected.

**Figure 12:**
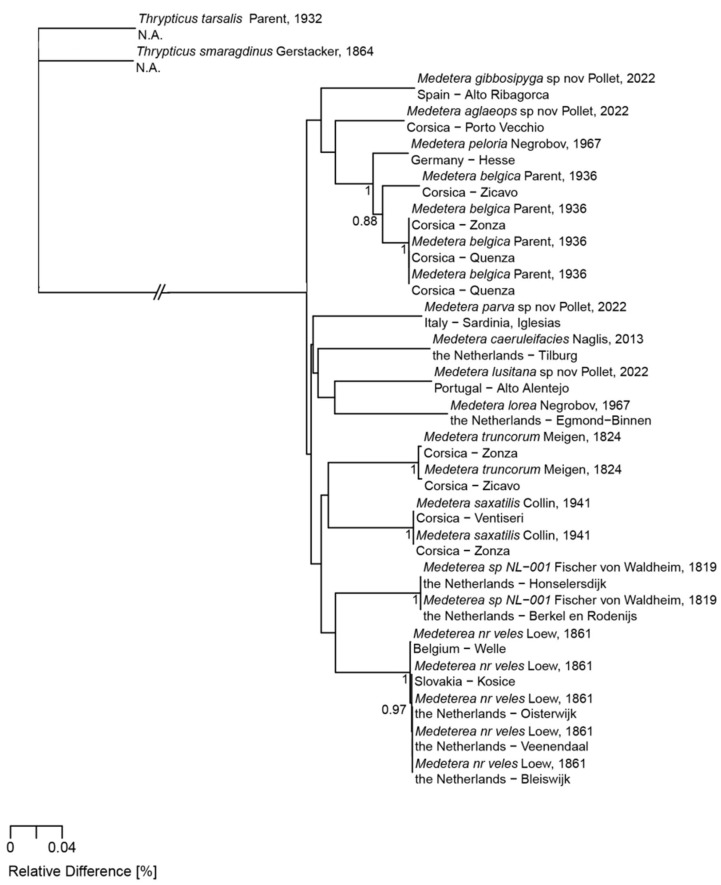
Cladogram of a selection of Palaearctic *Medetera* species on the basis of BOLD Systems sequences, including four of the new species described in this paper.

Based on COI sequences in BOLD Systems (see Figure 12), *M. aglaops* sp. nov., *M. gibbosipyga* sp. nov., *M. lusitana* sp. nov., and *M. parva* sp. nov. seem to belong to the *Medetera apicalis* species group *sensu* Bickel [12]. More specifically, they best fit the strongly supported *M. muralis* subclade *sensu* Pollet, Germann and Bernasconi [13], thus far only comprising *M. muralis* Meigen, 1824 and *M. belgica* Parent, 1936. Both latter species lack the ad–pd bristle pair of the mid tibia, which was thus far considered of great phylogenetic significance. However, shared characters in genital appendages, in particular the shape of the hypandrium and cercus, suggest that all seven species described here are closely related and even form a distinct lineage within the *M. apicalis* species group. Indeed, other species of the latter species group show a cercus with a distinctly different bristle arrangement and shapes, and quite often a hypandrium with a terminal enlargement (see [31,32,33]). This seems to imply that the absence or presence of the ad–pd bristle pair might not be as phylogenetically relevant as initially anticipated. The same holds true for acrostichal bristles on the mesonotum which were always present in four species, always lacking in two species, and of a variable status in one species.

Moreover, from an ecological perspective, most of the new species differ from most species of the *M. apicalis* species group, members of which are most often found on trunks of deciduous trees. As all specimens of *M. corsicana* sp. nov. and *M. parva* sp. nov. originate from Malaise traps samples, no information on their (micro)habitat preference is available. However, all five remaining species were mainly encountered in rather dry biotopes, with sclerophyllous vegetation types and/or open (often coniferous) forest where they mostly occurred on hard, rocky substrates. As pointed out previously [13], this behaviour is also observed in *M. muralis* and *M. belgica* which further supports their phylogenetic relationship.

The question remains why these species have been discovered only recently despite their conspicuous eye colour pattern. Species featuring eyes with different colour zones are known in Diaphorinae, e.g., *Cryptophleps* Lichtwardt, 1898 and *Diaphorus* Meigen, 1824, but the species described here are the first recorded in the genus *Medetera*. The first collected specimen (*M. aglaops* sp. nov.) in our data set dates back from 2004 (CONECOFOR survey, Sardinia), but a steady influx of collected specimens has been reported in the past ten years or so. Pictures of Portuguese and Spanish species (even with the head in frontal view) have been available on the Spanish citizen science biodiversity portal Biodiversidad Virtual ([34]; check for pictures of *Medetera*) for some time, but without any attempt to explore them further. The aim of this portal is to gather data on the distribution of mainly Spanish species via the registration of photographs of specimens by citizen scientists, and subsequent identification by experts. The reason for this situation seems to be a combination of several factors. First of all, dry habitat types where most of these species thrive are not among the standard sites visited and investigated by dolichopodid workers. Moreover, those interested in *Medetera* most often use Malaise traps or actively inspect tree trunks for specimens. As a result, these biotopes remain largely overlooked. Secondly, not many naturalists or Diptera workers in Europe are actively involved in observing and/or collecting dolichopodids. Moreover, even if conspicuous species are detected, at most a handful of taxonomic specialists in Europe are capable of properly treating them. This might also be considered part of the taxonomic impediment or Linnean shortfall [35]. Thirdly, most of the latter experts are not employed as entomologists and carry out their research in their spare time, which inevitably leads to delays. Finally, *Medetera* is undoubtedly one of the most challenging genera to study, in part due to the lack of distinct diagnostic features. It thus takes quite a lot of effort and time to verify whether a species is new to science or not, which often includes the examination of type specimens and/or comparison with specimens from reference collections.

## 5. Conclusions

This paper represents an important addition to the knowledge on a previously underexplored part of *Medetera* diversity in Europe. With this publication, we hope to encourage both Diptera workers and general naturalists to keep an eye open for these insects or search for them actively during their upcoming (collecting) trips. In this way, our knowledge on their distribution and ecology could increase substantially and possibly even additional species new to science might be detected. In this respect, blue or other dark coloured pan traps might be a promising collecting tool. Indeed, *Medetera* are most attracted to this colour range, regardless of whether tree-trunk dwelling or xerophilous species are concerned. Moreover, pan traps can produce fair yields in less than one hour [36].

## Data Availability

The data presented in this study i.e., detailed distribution records of the 7 described species including habitat descriptions, are openly available as dataset in GBIF at https://doi.org/10.15468/s8c7n9.
